# Community interventions for pandemic preparedness: A scoping review of pandemic preparedness lessons from HIV, COVID-19, and other public health emergencies of international concern

**DOI:** 10.1371/journal.pgph.0002758

**Published:** 2024-05-06

**Authors:** Sali Hafez, Sharif A. Ismail, Zandile Zibwowa, Nadin Alhamshary, Reem Elsayed, Mandeep Dhaliwal, Fiona Samuels, Ade Fakoya

**Affiliations:** 1 Faculty of Public Health and Policy, London School of Hygiene and Tropical Medicine, London, United Kingdom; 2 The Nuffield Centre for International Health and Development, School of Medicine, The University of Leeds, Leeds, United Kingdom; 3 The University of Western Cape, Cape Town, South Africa; 4 HIV and Health Group, United Nations Development Program, New York, United States of America; 5 Centre for Public Health and Policy, Queen Mary University of London, London, United Kingdom; 6 Institute for Global Health, University College London, London, United Kingdom; Dr. D.Y. Patil Medical College, Hospital and Research Centre, Dr. D.Y. Patil Vidyapeeth, Pune, INDIA

## Abstract

Community action is broadly recognised as central to comprehensive and effective system responses to pandemics. However, there is uncertainty about how and where communities can be best supported to bolster long-term resilience and preparedness. We applied a typology of community interventions (Community Informing, Consulting, Involving, Collaborating or Empowering–or CICICE) to cover the diverse range of interventions identified across the literature and used this to structure a scoping review addressing three linked topics: (i) how CICICE interventions have been understood and applied in the literature on epidemic and pandemic preparedness; (ii) the spectrum of interventions that have been implemented to strengthen CICICE and (iii) what evidence is available on their effectiveness in influencing preparedness for current and future emergencies. We drew on peer-reviewed and grey literature from the HIV (from 2000) and COVID-19 pandemics and recent public health emergencies of international concern (from 2008), identified through systematic searches in MEDLINE, Scopus, the Cochrane Collaboration database, supplemented by keyword-structured searches in GoogleScholar and websites of relevant global health organisations. Following screening and extraction, key themes were identified using a combined inductive/deductive approach. 130 papers met the criteria for inclusion. Interventions for preparedness were identified across the spectrum of CICICE. Most work on COVID-19 focused on informing and consulting rather than capacity building and empowerment. The literature on HIV was more likely to report interventions emphasising human rights perspectives and empowerment. There was little robust evidence on the role of CICICE interventions in building preparedness. Evidence of effect was most robust for multi-component interventions for HIV prevention and control. Much of the reporting focused on intermediate outcomes, including measures of health service utilisation. We put forward a series of recommendations to help address evidence shortfalls, including clarifying definitions, organising and stratifying interventions by several parameters and strengthening evaluation methods for CICICE.

## Introduction

There is widespread recognition of the growing threat of infectious disease epidemics and pandemics spreading across countries or continents—especially following the recent Ebola and COVID-19 pandemics. Vulnerable groups (including children, older adults, ethnic minorities and other at-risk groups) have disproportionately borne the brunt of significant health, social, and economic effects of these epidemics and pandemics, with varying degrees of support received depending on the context. While many argue that communities should or need to play a critical role in supporting and leading preparedness and response efforts, work still needs to be done to engage them effectively. In many instances, modes of engagement with communities—especially by state authorities–have come too late, often as an afterthought are not adequately thought through and have undermined public trust in and support for, disease prevention and control measures [1, 2].

Better community engagement is essential for tackling misinformation, bolstering trust, building social cohesion, and improving long-term health outcomes and equity in responses. Experiences from the historical and ongoing HIV response have shown an evolution of the role of communities in response and preparedness efforts, particularly when therapeutic options were limited [3]. The West African Ebola epidemic of 2014–16 prompted extensive reviews of the limitations of national and international response mechanisms wherein inadequate community engagement and participation in decision-making, programme implementation, and service delivery featured prominently [4–7].

Although many authors have called for greater involvement of community and community groups in disease prevention and control (e.g. [8]) challenges to meaningful involvement have repeatedly arisen in responses to past epidemics [9, 10]. Experiences during the COVID-19 pandemic were similarly limited: as the pandemic progressed, efforts were made to better align work on prevention and control with what was known from experience but often as an afterthought [11]. Partly, this is down to uncertainty as to what meaningful community involvement means [12]. There is also a limited understanding of which community interventions are most effective in strengthening epidemic and pandemic preparedness and how, where and with whom these are optimally provided. For example, risk communication is widely acknowledged as an essential element of community engagement and is often at the centre of community engagement work as articulated in key policies [13]. However, RCCE represents just one component of a much broader set of actions that must be supported to optimise preparedness and response, including empowerment of communities. These necessary broader actions are often not recognised in documents and key policies [14–16].

A central function of this review is to consider what lessons we might learn from past community engagement, participation and empowerment work. By drawing upon experiences from significant global health emergencies, including HIV, COVID-19 and other public health emergencies of international concern (PHEICs) over the past 15 years (Ebola, MERS, Zika and SARS-CoV-1), we seek to identify and analyse key factors that can contribute to effective future preparedness strategies, helping to improve overall system resilience [10].

In this review, we focus on community engagement and empowerment actions, which may increase capacity to deal with current and future shocks from epidemics or pandemics. Our review considers factors and interventions that could directly contribute to improved preparedness rather than the responses to the epidemics and pandemics themselves. However, learning from past emergencies inevitably influences preparedness to some degree, so these dividing lines are not absolute. We define preparedness in this context as “the knowledge, capacity and organisational systems that governments, response and recovery organisations, communities, and individuals develop to anticipate, respond to, or recover from infectious disease epidemics or pandemic-related emergencies” [17]. Reflecting on the contribution that it may make to preparedness; we also consider markers of community resilience–defined as a “community’s degree of adaptability to changing circumstances and challenges" [18]. In doing so, however, we recognise that resilience is a contested concept with interdisciplinary origins outside health, and the capacity to transform community relations in anticipation of shocks can be just as important as adaptation [19, 20].

We use the term CICICE (Community Information, Consultation, Involvement, Collaboration and Empowerment) to cover the diverse and complex range of approaches identified across the literature, as explained in the methods section below. In doing so, we drew on an existing categorisation of community approaches that is widely employed in the research literature on this topic [21–23].

We hypothesised that investments in CICICE can enhance the capacity to deal with current and future pandemics through various potential pathways (some of which are illustrated indicatively in Fig 2, but that investment is required along the spectrum from informing communities to supporting their empowerment. Among other mechanisms, CICICE activities can enhance this capacity by: improving access to (and ability to assimilate and correctly interpret) information on epidemic and pandemic threats, strengthening networks and the social capital required to help support community responses; helping to mobilise material resources at community level; and empowering communities themselves to make decisions on how and where money and other resources are used to support long-term resilience best.

We aimed to (i) understand how community interventions have been understood and applied in the literature on epidemic and pandemic preparedness; (ii) map the spectrum of interventions that have been implemented to strengthen community preparedness for future epidemics and pandemics; and (iii) map evidence on the contribution of these in influencing preparedness for current and future infectious disease emergencies, where this is available. Because of the exploratory nature of the review questions and the emphasis on clarification of concepts, we applied a scoping review methodology.

This study contributes to the body of knowledge on community-centred strategies for pandemic preparedness. Our intention is to help inform and shape future policy and practice by highlighting effective strategies and interventions to promote community engagement and empowerment for better preparedness.

## Methods

**Fig 1:**
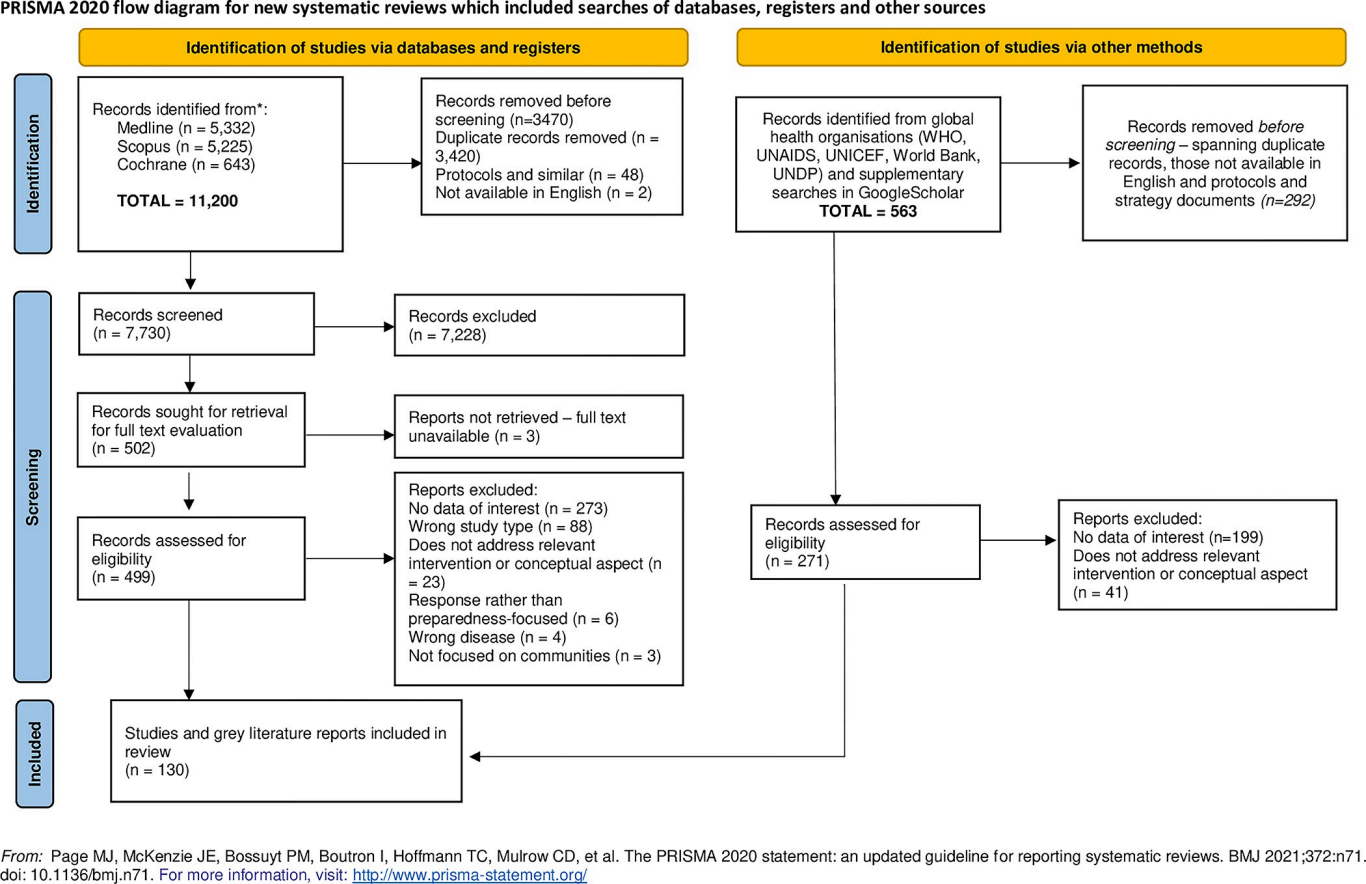
PRISMA flowchart describing the article screening process.

This was a scoping review of relevant peer-reviewed and grey literature, drawing on principles set out by the Joanna Briggs Institute [24, 25]. Protocol details including the PICO formulation for the review can be found in Table A of **S1 Text**. Findings are reported here broadly in accordance with PRISMA-ScR guidance [26], as indicated in Fig 1 and **S1 Checklist**.

**Fig 2:**
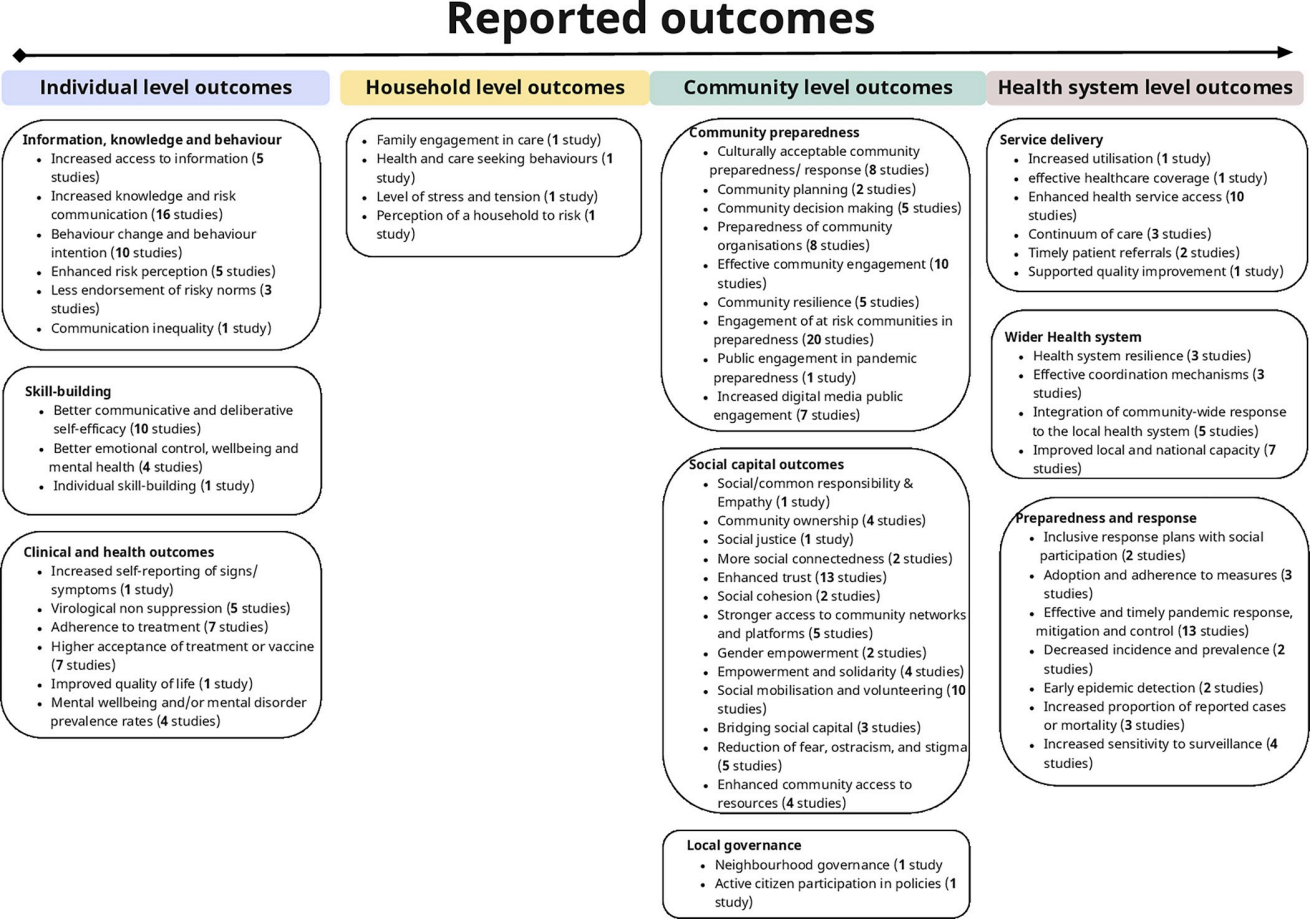
The working theory of change for CICICE activities informing this analysis. The review focuses on measures contributing to directly improved preparedness in anticipation of epidemics and pandemics. However, we acknowledge the importance of feedback and learning (represented here by a feedback loop) from response work.

In conceptualising how CICICE activities might ultimately contribute to improved preparedness, we developed a working theory of change setting out a range of spaces for action and a range of high-level intervention mechanisms by which this might occur Fig 2.

### Conceptualising community interventions

Definitions of “community”, “community engagement”, and allied terms vary greatly [12]. For this review, we developed and used the term CICICE to encompass the full range of activities that may occur in this space, from simply informing communities (e.g. about epidemic or pandemic risks) to empowering them to act independently to support preparedness. We drew on an extensive research literature on this topic [21–23]. One important goal of this review is to help bring greater conceptual clarity regarding the full range of intervention types, spanning risk communication measures (such as public information campaigns and social media messaging), community outreach work, peer-to-peer interventions through to community-led service delivery and related approaches such as community-based epidemic surveillance and monitoring (see Table 1).

**Table 1:** The spectrum of CICICE approaches considered in this scoping review, adapted from [21–23].

*Type*	*Informing*	*Consulting*	*Involving*	*Collaborating*	*Empowering*
Description	Information flow primarily in one direction (to inform community members), leading to establishment of communication and outreach channels	Communication to the community and then back, to seek feedback (e.g. on content), leading to strengthened networks	Bidirectional communication and cooperation, leading to partnership formation between communities and decision-makers	Bidirectional communication with partnership formation with community members on project/ intervention development, implementation and evaluation, leading to partnership development and greater trust between communities and decision-makers	Bidirectional relationship formation with active community leadership, leading to strong relationships of trust and, potentially, transformation of power relations in favour of communities
Example interventions	Simple risk communication measures e.g. issuing leaflets or use of social media platforms to support dissemination of risk information [27]	Advanced risk communication and community engagement measures in which products are co-developed with community members [28]; SMS surveys for two-way communication and dissemination of risk information [29]; use of mobile technology to support capacity and awareness among informal workers [30]	Community-based health workforce development (e.g. CHWs [31, 32]; community champions(	Community based surveillance supported by CHWs [33]; collaboration with faith-based organisations to support community engagement [34], or use of community influencers [35]	Peer-to-peer education work and skills development [36]; stigma reduction and confidence-building measures [37]

Implicit within this framework is an acknowledgement that community interventions may influence, to greater or lesser degrees, both the agency that communities have, and as a corollary of this, the extent to which power relations negatively affecting community members are altered. Interventions supporting empowerment are likely to focus on high degrees of community agency and may have transformative effects on power relations [38].

### Outcomes

We considered a wide range of outcomes encompassing a comprehensive range of diverse indicators across five outcome domains: individual (such as increased knowledge or behaviour change), household (such as empowerment, and wellbeing of household members), community (such as social capital or trust), health systems outcomes (such as health service utilisation) and ultimately health outcomes (such as infection-related mortality or morbidity).

### Search strategy

We considered literature on COVID-19 and other PHEICs (Ebola, MERS, SARS-CoV-1 and Zika) published between 1st January 2008 and 4^th^ April 2023, as explained in **S1 Text**. We also searched for literature relating to HIV/AIDS, but in view of the duration of that epidemic, extended the beginning of our search period back to 1st January 2000 to capture the longer-term evolution of thinking on CICICE in the context of the HIV response given the duration of that pandemic.

We performed systematic searches in MEDLINE, Scopus and the Cochrane Collaboration databases, augmented by keyword-structured searches in GoogleScholar and targeted searches of websites for key relevant organisations in global health–namely the WHO, UNICEF, UNAIDS, UNDP and the World Bank. We included searches of these five institutions because they are key actors involved in developing technical guidance documents on community engagement (including in pandemics). We also searched for literature from key global initiatives on pandemic preparedness that have emerged in recent years–namely the Global Preparedness Monitoring Board (GPMB) and the Independent Panel for Pandemic Preparedness and Response. Searches were structured using keywords in three main domains: terms describing community engagement and/or participation; those relating to key diseases of interest; and those describing the critical outcomes of interest for our analysis, namely epidemic/pandemic preparedness, and community resilience (please see Box A of **S1 Text** for a sample application of the search strategy). We also looked at studies addressing a range of secondary outcomes including health service utilisation, disease outcomes (mortality, morbidity and–for diseases such as HIV, treatment response measures), and proxy markers for resilience such as measures of community cohesion. Precise outcome definitions applied varied according to the study.

### Screening and extraction

After de-duplication, studies identified through the searches were screened for inclusion by three members of the research team (SAI, SH, ZZ), initially on title/abstract and then on full text. We included primary studies reporting results relevant to the outcomes given above, spanning observational, experimental, and mixed-methods study designs but excluding case studies and research letters (**S1 Text**). We also included reviews (narrative and systematic). Finally, where commentaries or editorials identified by the searches explicitly reported conceptual frameworks or provided theoretical insights with relevance to the CICICE conceptualisation, these were included in our analysis to help address the first of the review aims set out in the Introduction. Technical guidance documents from normative institutions were also included on the basis that these included recommendations derived from evidence syntheses and/or expert consensus exercises.

Relevant material from included studies was extracted using a pre-developed, MS Excel template by five members of the research team, working independently (SAI, SH, ZZ, RE and NE). Extraction focused on key study characteristics (e.g. date of publication, location), definitions of “community” and related terms applied, interventions described (where relevant), outcomes addressed and–where reported–intervention effects. In view of the short turnaround for this work, an audit of extracts for a randomly selected 5% sample of included papers was conducted to ensure the accuracy of extraction rather than full extraction in duplicate.

### Analysis

Data extracted were reviewed by three members of the study team (SAI, SH and ZZ) to identify common themes using a combined inductive/deductive approach drawing on the conceptual framework given in Fig 2 (the charting step) [24, 25]. Following a whole-team workshop to review preliminary results, findings across all studies were narratively synthesised. To help interpret the range of CICICE approaches addressed in the literature, we assessed which of the approaches (i.e. informing, consulting, involving, collaborating, empowering) each study in the review considered. This analysis was performed only primary research studies, and not for review articles as these by definition included multiple intervention types concurrently. Because of the diversity of sources consulted, and the dual conceptual-analytical orientation of the review, we did not carry out formal critical appraisal of included sources. However, in the results below we emphasise key features such as study design and major limitations to help weight reporting.

## Results

**Fig 3:**
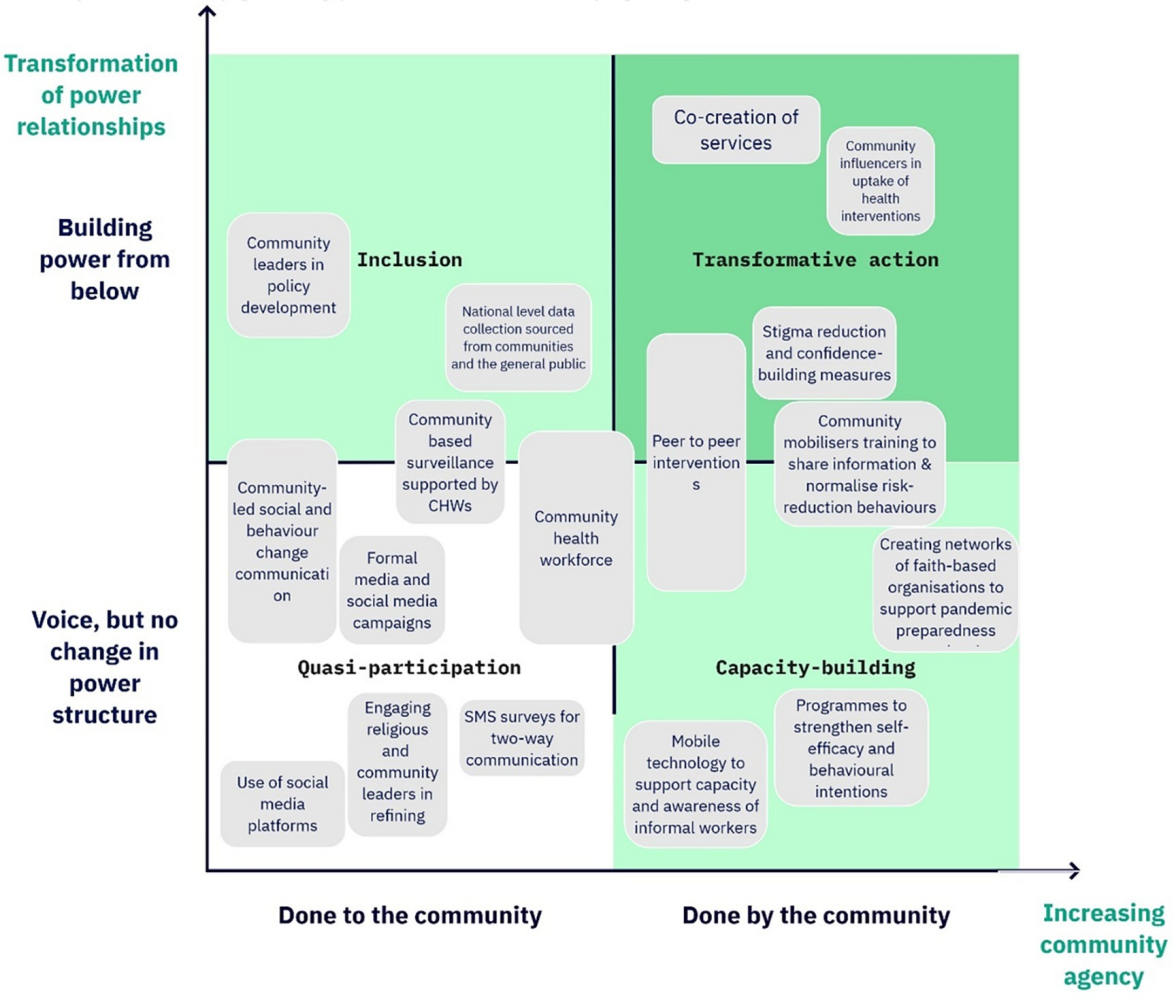
Distribution of studies included by country in which the analysis was set. Those labelled “multiple” included reviews (systematic and narrative), comparative studies, and guidance documents issued by selected global health organisations.

We included a total of n = 130 studies, of which n = 102 were peer-reviewed articles and n = 28 were grey literature reports. Just over a third of the included sources (n = 47, 36%) focused on COVID-19; n = 33 studies were drawn from the HIV/AIDS literature, n = 24 addressed PHEICs of different types, and the remaining 26 sources covered multiple diseases. Included sources spanned many different setting types and populations. Many studies (n = 66) addressed multiple settings concurrently. The United States was the most common individual research setting (n = 15), with n = 5 studies from Liberia and Sierra Leone building on experiences during the West African Ebola epidemic (Fig 3).

Much of the material included was contemporary: around 70% of the studies (n = 93) had been published within the past five years. Material on HIV was–in line with the search strategy adopted for the review–distributed over a broader time period although 58% (n = 19) of included studies on this disease were published within the past five years.

Research designs among included studies were diverse. The largest single class of studies were narrative reviews (n = 31, 24%), but we also included n = 20 systematic and scoping reviews, n = 16 trials and quasi-experimental studies, and n = 14 mixed methods studies. 22 guidance documents from selected global health organisations were included.

A flowchart summarising the search and screening process is given in Fig 1. A paper-by-paper summary of results is provided in **S1 Table**.

### The scope of CICICE in infectious disease epidemics or pandemic-related emergencies

#### Meanings of “community”

To understand how CICICE might contribute to epidemic and pandemic preparedness, clarity on the meanings of “community” and “engagement” as well as allied terms such as “communication”, “participation” and “empowerment” is needed. Most included studies (n = 93, 72%) gave no formal definition of community. Among those that did offer a definition, we noted a conceptual division between studies that adopted epidemiological and/or public health-oriented perspectives, and those that took one rooted more clearly in the social sciences. Studies in the former category typically defined “community” in terms of specific population groups—whether based on gender [39], occupation [36, 40], vulnerable group status (e.g. sex workers) [41], geographically-defined (e.g. rural or urban communities or those living in specific localities), linked to socio-economic status [42, 43], specific age category [44], or according to perceived vulnerability (e.g. urban poor) [42]. Studies in the latter group engaged more closely with aspects such as identity and belonging that might shape the cohesion of distinct communities (as understood by community members themselves) [45]. These studies also recognised that drawing community boundaries is a subjective process and the way in which external actors, including public health practitioners, define these boundaries might not accord with the views of community members themselves [46]. Finally, some studies, in addressing the role of identity and belonging, also highlighted that people living in a specific geography might not identify with a community defined in that way, and may understand community instead as either a collection of individuals who share similar interests and identities, or as a group with designated characteristics or identifiably “at-risk” [45].

#### Definitions of CICICE terms

Language used to describe CICICE was varied and terms such as “community engagement”, “community participation”, “community development”, “capacity building” and others were often used interchangeably. At one end of this spectrum were definitions of community engagement that adopted a top-down perspective—e.g. “activities that build trust between all levels of government and its constituents” [18]. Elsewhere, definitions emphasised the importance of co-production—understanding community engagement as the “participation of individuals, groups, and structures within the social boundary in decision-making, planning, design, governance, and service delivery” [47, 48]. Building relationships as a way of facilitating work to address health issues and promote well-being was viewed as a key component of community engagement work irrespective of the approach [32, 49, 50].

A smaller number of papers reviewed considered community “empowerment”—overwhelmingly in the context of HIV/AIDS [40, 45, 51–54]. Definitions of empowerment–where given–emphasised community control, social justice and aspects such as community quality of life [55, 56]. In doing so, they distinguished the collective focus of empowerment from individual behavioural or cognitive interventions focused on self-efficacy [53, 55]. Gaining power and autonomy over decision-making–for populations to whom that power was otherwise denied–was seen as a central aspect of empowerment, especially in the HIV/AIDS literature [40].

Variations in definitions sometimes reflected differences in whether community engagement and empowerment were understood primarily in terms of the intended *outcomes* from this work, or whether it was about the *process* of developing relationships that might ultimately lead to better community preparedness and resilience [55, 57].

### Mapping interventions to support CICICE in the context of infectious disease epidemics or pandemic-related emergencies

A wide range of interventions were described in the literature. Many studies–especially those addressing HIV, and those published more recently–emphasised the importance of composite approaches rather than “silver bullet” interventions for CICICE (e.g. [23, 40]). Interventions described varied in the extent to which they focused on (i) specific populations or the population as a whole; (ii) specific places of intervention; and (iii) the pathway to outcomes and impact. For example, while a large majority of studies on COVID-19 addressed the general population, HIV studies frequently focused on interventions targeting specific groups identified as vulnerable [e.g. 37, 40]. They were also more likely to consider outreach approaches to CICICE in the contexts in which people live and work [36].

Table 2 maps out where interventions described in the n = 106 empirically-oriented studies included in this review were assessed by members of the research team to fall with respect to the spectrum of CICICE approaches outlined in Table 1 above. Most studies of this type addressed “inform” (n = 80), “consult” (n = 85) or “involve” (n = 75) interventions; n = 25 addressed interventions aiming to directly empower participants. This pattern also varied by disease: of n = 37 papers focusing on COVID-19, n = 5 (14%) described interventions seeking to empower participants; for HIV, the equivalent figure was 17/31 (55%).

**Table 2:** Heatmap visualising the spread of CICICE interventions for each disease area for intervention studies included in the review, using the conceptual approach outlined in Fig 2. Green represents a low number of studies, with an increasing intensity marked by the progression to dark green, yellow, orange, and finally red as the number of studies increases.

*Authors*	*Year*	*Broad disease focus*	*Inform*	*Consult*	*Involve*	*Collaborate*	*Empower*
Abdalla et al [58]	2021	COVID-19					
Aung et al [59]	2021	COVID-19					
Junior and Morais [45]	2020	COVID-19					
Brewer et al [60]	2020	COVID-19					
Choukou et al [61]	2022	COVID-19					
Cruwys et al [62]	2022	COVID-19					
den Broeder et al [63]	2022	COVID-19					
Fransen et al [64]	2022	COVID-19					
Kalocsányiová et al [65]	2022	COVID-19					
Lau [66]	2020	COVID-19					
Lim and Nakazato [67]	2020	COVID-19					
Maher and Murphet [68]	2020	COVID-19					
Mahmud et al [69]	2021	COVID-19					
Malik et al [70]	2021	COVID-19					
Mat Dawi et al [71]	2021	COVID-19					
Nöstlinger et al [72]	2022	COVID-19					
Rämgård et al [73]	2023	COVID-19					
Rashmi and Lekshmi [74]	2021	COVID-19					
Rezaei et al [75]	2022	COVID-19					
Sahoo et al [42]	2023	COVID-19					
Wild et al [76]	2021	COVID-19					
Tambo et al [77]	2021	COVID-19					
WHO [78]	2022	COVID-19					
WHO [79]	2022	COVID-19					
IFRC, UNICEF, WHO [80]	2020	COVID-19					
UNICEF, WHO [81]	2021	COVID-19					
IFRC, UNICEF [82]	2020	COVID-19					
IFRC, UNICEF, WHO [83]	2020	COVID-19					
GOARN, IFRC, UNICEF, WHO [84]	2021	COVID-19					
IFRC, UNICEF, WHO EMRO [57]	2020	COVID-19					
WHO Europe [85]	2021	COVID-19					
UNICEF, WHO Europe [86]	2022	COVID-19					
WHO [87]	2020	COVID-19					
WHO [88]	2020	COVID-19					
WHO Europe [89]	2022	COVID-19					
WHO Western Pacific Region [90]	2020	COVID-19					
UNDP [91]	2020	COVID-19					
Haberer et al [92]	2021	HIV					
Muriisa and Jamil [56]	2011	HIV					
Akeju et al [93]	2021	HIV					
Bauman et al [44]	2021	HIV					
Beattie et al [40]	2014	HIV					
Carballo-Dieguez et al [53]	2005	HIV					
Carbone et al [94]	2019	HIV					
Carlson et al [95]	2012	HIV					
Choi et al [96]	2022	HIV					
Cohen et al [43]	2022	HIV					
Dunbar et al [54]	2020	HIV					
Feyissa et al [97]	2019	HIV					
Gulaid and Kiragu [28]	2012	HIV					
Harrison [98]	2019	HIV					
Hickey et al [99]	2015	HIV					
Mayo-Wilson et al [100]	2020	HIV					
Kerrigan et al [101]	2017	HIV					
Kerrigan et al [55]	2013	HIV					
Kiragu et al [41]	2020	HIV					
Li et al [102]	2022	HIV					
Magidson et al [103]	2022	HIV					
Lin Miller et al [104]	2017	HIV					
Moore et al [105]	2022	HIV					
Mwai et al [32]	2013	HIV					
Newman et al [35]	2022	HIV					
Remme et al [39]	2014	HIV					
Sevelius et al [37]	2022	HIV					
Simms et al [106]	2022	HIV					
Wilson et al [36]	2019	HIV					
Abramsky et al [107]	2014	HIV					
UNDP [108]	2012	HIV					
WHO [109]	2018	Multiple					
Chiam et al [110]	2022	Multiple					
Mohammadpour et al [111]	2021	Multiple					
Cummings et al [112]	2019	Multiple					
Obregon et al [113]	2020	Multiple					
Osborne et al [51]	2021	Multiple					
Schwartz and Yen [114]	2017	Multiple					
Collins et al [10]	2023	Multiple					
Cook and Seymour [115]	2013	Multiple					
Ernawati et al [116]	2020	Multiple					
Iyiani et al [117]	2011	Multiple					
McGowan et al [118]	2022	Multiple					
Olowu [119]	2015	Multiple					
Wroe et al [31]	2021	Multiple					
Abramowitz et al [120]	2018	PHEIC					
Armstrong-Mensah and Ndiaye [33]	2018	PHEIC					
Barker et al [121]	2020	PHEIC					
Bouye et al [122]	2009	PHEIC					
Frimpong et al [123]	2023	PHEIC					
Kiser and Lovelace [124]	2019	PHEIC					
Lwin et al [27]	2018	PHEIC					
Mase et al [125]	2017	PHEIC					
Masotti et al [126]	2013	PHEIC					
Mayhew et al [4]	2021	PHEIC					
Meyer et al [127]	2018	PHEIC					
Miller et al [128]	2018	PHEIC					
Ndiaye et al [129]	2014	PHEIC					
Nsubuga et al [130]	2021	PHEIC					
Olu et al [131]	2016	PHEIC					
Simen-Kapeu et al [132]	2021	PHEIC					
Skrip et al [52]	2020	PHEIC					
Wilkinson et al [46]	2017	PHEIC					
WHO [133]	2022	PHEIC					
IFRC, UNICEF, WHO [134]	2018	PHEIC					
Singaravelu et al [135]	2019	PHEIC					
UNICEF, WHO [136]	2014	PHEIC					
			80	85	75	57	25

Measures to support risk communication featured prominently in the literature, including the use of media campaigns, social media tools, digital tools (notwithstanding issues of variable access especially among vulnerable populations [61] and even legislation to combat the spread of misinformation [58]—all ultimately with the aim of building trust and engagement [61, 109]. The focus of much of this literature was on what public authorities (the “state”) can do to improve knowledge and awareness among communities. More recent work addressed messengers (e.g. community or religious leaders) and channels for information delivery (e.g. written materials, social media channels), as well as the content of the “message” itself. Partnerships with faith leaders, indigenous community leaders, and community health partnerships in delivering risk messaging were identified as ways of improving community receptivity, especially where messages have been co-created with these groups [65]. Engagement with traditional healers as part of risk communication and community engagement (RCCE) work was also described in the recent Ebola pandemic in the Democratic Republic of Congo [134, 135].

Literature, especially on Ebola epidemic responses, highlighted the role of trust-building through community dialogue and dedicated liaison work by, for example, community volunteers [4, 123, 135]. Trust is to some extent linked to public health messaging and the way in which it is delivered; a body of work on Ebola, for example, has shown how the tendency by public health authorities to communicate prevention messages in written form (when many living in affected areas are illiterate) or in official languages (not always spoken locally) can undermine trust at community level [4].

Community-based surveillance and service delivery was addressed by a small number of studies, most of which focused on the role of community health workers (CHWs). CHWs may have an important bridging role in supporting community inclusion and capacity building—especially as members of affected communities themselves [31, 32]. The range of CHW functions described was diverse, including formal delivery of health services, through to counselling and care navigation (in directing people to other sources of support) [32, 69]. However, wider factors such as the supervision and training of community-based surveillance workers, and the extent of integration with other surveillance systems also appear to be important in determining the success of these approaches [118].

A smaller body of work considered the role of interventions to build neighbourhood ties (to promote social capital), to promote volunteerism, the need for support for community organisations that often provide the backbone for community responses in the event of an epidemic or pandemic, and other measures. Mechanisms for doing so were diverse and often composite, comprising combinations of peer-to-peer work, innovative use of technology and other approaches [63]. Peer-to-peer work and self-reflection were identified as important, for example, for reducing the stigma attached to epidemics particularly HIV) as a means for improving the effectiveness of prevention [54, 97].

A small set of papers considered the role that activism can play in positively shifting norms around community empowerment for better epidemic and pandemic preparedness. This was an important focus in the HIV literature—in consideration of marginalised groups for whom social structures have proven barriers to effective disease prevention and control work, such as sex workers [55]. The importance of activism in addressing the politicisation of certain aspects of the COVID-19 response emerged as a key theme from some of this literature [92].

More recent studies on community engagement and empowerment typically focused on multi-component CICICE interventions blending one or more of the categories of action outlined above. This was especially the case for those communities identified as vulnerable and especially at greater risk from HIV [41, 107]. Intervention packages included conventional health modalities such as community-based disease surveillance and/or the delivery of community health services. These intervention packages also included action on the wider social determinants of health, including food distribution, support for improved access to water, sanitation and hygiene, and financial support for the most vulnerable, including through micro-enterprise promotion and cash transfers [39, 42].

**Fig 4:**
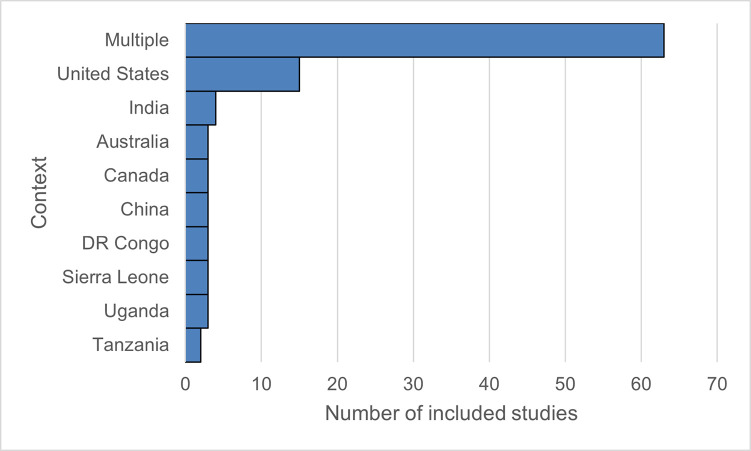
Mapping the diversity of literature on interventions within the realm of CICICE interventions for epidemic preparedness along two dimensions: The extent to which communities develop true agency (x-axis); and the degree to which underlying power relations are transformed (y-axis). Titles in the four quadrants identified how far along a pathway to transformation each form of intervention lies. In this visualisation, individual studies are mapping according to where they fall; systematic reviews and scoping reviews are necessarily excluded because they typically included interventions of many different types (Adapted from [39]).

Given the range of intervention categories in this review, the diversity of putative intervention mechanisms for bringing about change was similarly broad. Drawing on an approach outlined by Cole *et al* [38], Fig 4 maps the range of interventions described along two categories: the extent to which each intervention built community agency; and the extent to which it resulted in changes in power relations. Many intervention types were applied differently across studies with implications for their effects on transformation of power relations, or on community agency. Peer-led interventions might promote capacity-building, for example [94], but could have transformative effects if embedded within wider packages of interventions to support community empowerment [55]. Studies included in the review predominantly fell into the bottom left-hand quadrant in Fig 4 (quasi-participation). We identified few studies where the stated objective of the intervention was to promote transformative action; almost all relevant studies in this group were from the HIV literature.

### Outcomes and effectiveness of interventions in influencing preparedness capacities

**Fig 5:**
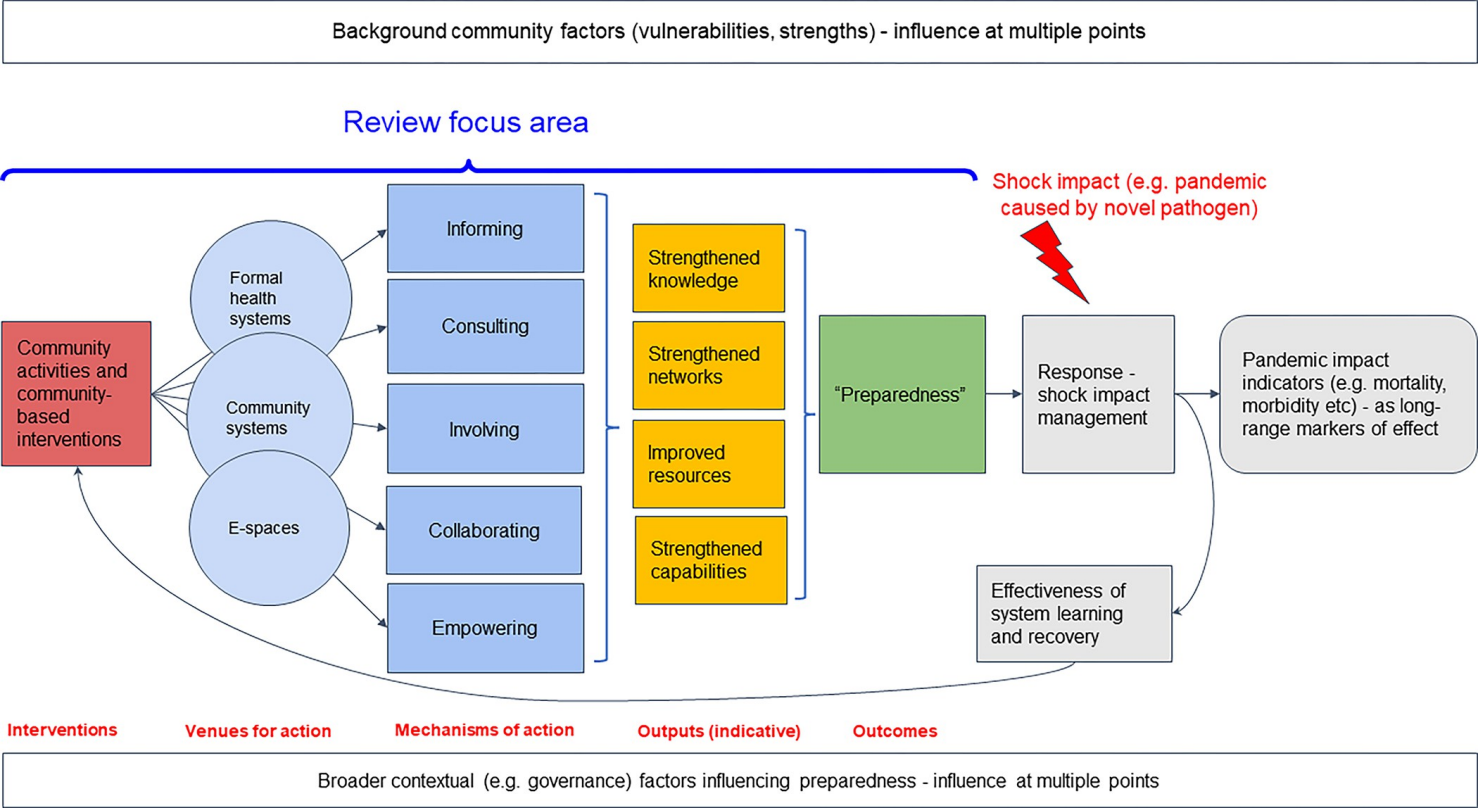
The spectrum of outcomes reported in studies included in the review, ranging from those framed at individual level, through households to communities and finally health system level. Included studies frequently addressed more than one of these outcomes concurrently, though the intervention described was framed at community level (to meet the inclusion criteria for the review). Numbers of studies addressing each outcome are given in brackets.

We identified a broad range of outcomes described in included studies, ranging from individual level to whole health system effects (Fig 5). Few studies dealt directly with “preparedness” as a measurable outcome [111, 137], reporting instead proxy measures for different aspects of preparedness. Studies drawn from epidemiology or public health overwhelmingly considered intermediate markers such as increased health service utilisation or occasionally, health outcomes (e.g. mortality, morbidity and–for HIV–metrics such as viral load [62]). Literature from the social and behavioural sciences considered outcomes such as labour market recruitment and retention or confidence in government-run health services more linked to community resilience [120].

Outcome reporting in the large body of work on risk communication was predominantly focused at the individual level, but the quality of reporting was variable. As the authors of one systematic review noted, "how [different] messaging [strategies] impacted vulnerable communities’ risk and efficacy perceptions and actual behaviour (e.g., compliance with protective measures) was largely unexplored, leaving somewhat in doubt the effect these studies have had" [65]. Most risk communication studies reported outcomes such as increased knowledge, enhanced risk perception or changed behaviour at the individual level (e.g. [44, 63]).

At the community level, effects seen in community capacity development and empowerment were again variable according to the specific design of the intervention. Measures to improve local social capital among people living with HIV in Kenya, for example, showed promising effects on intermediate outcomes such as health service utilisation and medication adherence [99]. Empowerment-focused interventions—such as those aiming to support livelihoods for better long-term community resilience—showed variable effects according to the outcome. For example, a well-conducted randomised controlled trial (RCT) of an agricultural intervention in rural Kenya showed some effects on broader health outcomes, including rates of depression among participants and improved agricultural productivity, but no statistically significant effects on HIV-related outcomes [43].

Multi-component, community empowerment-oriented interventions—almost all of which focused on HIV/AIDS—considered a broad spectrum of outcomes, including effects on improving self-efficacy, reducing stigma, improvements in behavioural prevention markers such as condom use, and disease outcome markers such as HIV incidence. Rigorously conducted RCTs of multi-component interventions in populations affected by HIV demonstrated improvements in self-efficacy (self-rated) across a diverse range of settings ranging from adolescents in Kenya [95] to young people in the urban United States [46]. A number of reviews showed positive effects from multi-component interventions that spanned improvements in access with community mobilisation and strategies designed to address harmful power relations for marginalised groups such as sex workers [55]. However, the extent to which these interventions translated into demonstrable changes in power relations at a macro-level (e.g. through demand for rights that were subsequently realised) was unclear [41].

We identified a small number of studies addressing community cohesion and aspects of social belonging as precursors to better preparedness and resilience. One evaluation, addressing social and neighbourhood identity as assets predisposing to greater resilience in the context of COVID-19, showed statistically significant differences in markers of well-being and reducing psychological distress among populations living in areas that had participated in annual social capital-building “neighbour days” prior to the pandemic, by comparison with those that had not [62].

At the health system level, work on community-based health service development—principally the use of CHWs—focused on effects on intermediate outcomes (e.g. service utilisation), with generally positive effects. One RCT from rural Malawi noted reductions in default rates from HIV clinics post-introduction of a programme [31]. A mixed methods study from countries directly affected by the West African Ebola epidemic showed a better rebound in essential health service utilisation following the worst period of the epidemic in areas where CHWs worked [128]. A smaller body of work considered broader effects on outcomes linked to preparedness. For example, a multi-component intervention showed improvements in health outcomes for HIV (e.g. better treatment adherence, better viral suppression at six months following initiation of treatment), in markers of health service utilisation (such as patient flow in clinics) and on preparedness-oriented outcomes such as stigma and strengthened family and local social relations [32].

The few studies that considered the role of community-based surveillance (which often depends on the work of CHWs at the local level) noted increases in case reporting by comparison with passive surveillance systems (e.g. [37, 50]). However, these studies were mostly small-scale observational or pilot studies [33]. A single study from Bangladesh during the COVID-19 pandemic was conducted on a larger scale and supported the utility of community surveillance overall but noted high levels of noise in data, especially in the early phases of implementation [69].

Finally, we identified only one study (a systematic review) that considered the costs of CICICE—alongside a range of other measures to promote gender-responsiveness in disease prevention and control work for HIV [39]. This review—focused on measures to reduce inequalities in outcomes for HIV based on gender and considered various community-based interventions including peer-support, community mobilisation among female sex workers, and work in livelihoods (e.g. microenterprise support and poverty reduction measures such as cash transfers)—identified positive results only for community mobilisation for female sex workers at $YS13-19 per DALY averted [39].

## Discussion

The primary aim of this review was to understand the potential contribution of different community approaches, termed CICICE, to epidemic and pandemic preparedness. We identified very limited evidence directly addressing the connection between CICICE interventions and measurable improvements in preparedness (however defined). Studies overwhelmingly considered effects on intermediate outcomes (such as measures of individual knowledge of disease risks, or health service utilisation metrics). They were framed at levels ranging from the individual to whole health system levels–although the predominant focus was on individual- and community-level effects with a marked absence of evidence at [138] the household level.

Overall, the evidence we considered emphasised that no silver-bullet approach for strengthening preparedness through CICICE exists. There were some differences across diseases, as outlined below, but the limits on the number of identified papers prevented us from identifying regional differences. We noted a trend towards the use of multi-component interventions, especially for HIV prevention and control, in recent years. Although work on system resilience in the COVID-19 response highlights the extent to which resilience hinges on multi-systemic factors, including other vulnerabilities that may exist well outside the normal realm of activity of the health system [139], there was a limited translation of this recognition into the design of community CICICE interventions for COVID-19. The focus has been overwhelmingly on risk communication, with little work considering community empowerment.

We observed a much greater context specificity (restriction to specific populations or localities) in the HIV literature and, to some extent, Ebola than work on PHEICs and COVID-19. This is partly linked to the geographical focus of past epidemics and the populations involved [134, 135]. In the fast-spreading respiratory epidemics/pandemics such as COVID-19 or SARS, the tendency has been to adopt a national, population-wide approach rather than local adaptation of approaches.

For HIV/AIDS, there was also a markedly stronger emphasis on using “human rights” based approaches, addressing stigma and focusing on “key populations”. This led to a greater emphasis on empowerment and rights-building in this literature. This likely reflects the historical trajectory of the HIV epidemic and the central role of communities and civil society in driving advancements in preventing and tackling the infection. More recent papers emphasise cost-effective approaches and security in the context of preparedness.

The term “preparedness” is a relatively recently used concept in the context of epidemics and pandemics and did not feature strongly in the included HIV literature. Discussion of pandemic preparedness has become much more prominent following the West African Ebola epidemic, especially in the context of COVID-19.

The lack of positive evidence of effect of CICICE interventions on preparedness may partly be explained by ongoing uncertainty regarding definitions of key terms in the literature in the three main disease areas investigated in this review [23]. We found substantial variations in how concepts including “community”, “engagement” and “empowerment” were understood across the research literature, sometimes depending on the disciplinary traditions from which papers originated.

There is also debate concerning prerequisites for preparedness at the community level and indeed the objectives of CICICE per se. Although there is a broad acknowledgement that investment in community resilience is important for epidemic preparedness and response, mechanisms for doing so and for understanding CICICE intervention effects remain poorly understood [18]. The question of whether CICICE should be viewed as an outcome in and of itself or whether it is a utilitarian objective, resulting in improved health outcomes, is also a major point of tension in the literature [23]. Included studies addressed interventions mostly concerned with quasi-participation of communities: we noted little practical recognition of community agency, and minimal impact on power imbalances, both key elements of participation. In this respect, there were striking differences in the emphases of the research literature by disease. The experiences of many of the communities affected by HIV for example, seems to have spurred a greater focus on truly transformative action from below than has generally been the case for COVID-19 or the PHEICs considered in this review.

Differences in approaches to conceptualisation of community, and CICICE, contributed to challenges in identifying definitive effects of community-oriented interventions and policies–a task that is in any case difficult because causal pathways from intervention to outcome are complex and there may be significant time lags between the point of intervention and the identification of an effect. Where positive effects were seen in better quality evidence considered in this review, these mostly concerned service utilisation measures or intermediate outcomes such as self-efficacy or treatment results, rather than long-term impacts.

Finally, we found no evidence of the cost-effectiveness of CICICE for epidemic and pandemic preparedness, despite the long track record of research on this topic in the HIV literature. This is an important finding in and of itself, but also because key considerations identified elsewhere–for example, the sustainability of financing models for community organisations that may strongly influence their scope for action and their effectiveness–were not addressed by studies included in this review [2].

### Strengths and limitations of the review

Although other scoping reviews have been conducted on aspects of community engagement and empowerment for epidemic preparedness [18, 110], this is–to our knowledge–the first review to present a broad overview of all the domains within CICICE and evidence of effectiveness. A central contribution of this paper has been to chart the variety of ways in which these key concepts and related intervention strategies have been understood in the literature on HIV, COVID-19 and PHEICs as a precursor to building greater consensus among practitioners and researchers working in this space.

There are imitations to the comprehensiveness of this analysis, given the breadth of the scope employed, the short time period available for the review, and the comparatively limited range of databases consulted. We included only English-language studies, which may have meant that some important results were missed. We consulted grey literature reports from a restrictive number of global health organisations, with the possibility of having missed a body of work published by other sources. It was not practically possible to include literature from pandemic preparedness programs and project evaluations, commonly developed by civil society organisations in this review, given the time and resources available. This literature may include important insights into complex interventions to support community engagement and empowerment–including negative results (contributing to publication bias). Finally–and in keeping with conventional scoping review methods—we did not formally critically appraise included studies, although we provide general observations here on the quality of the literature examined.

### Policy, practice, and research recommendations

Our findings point to a series of research recommendations and suggest various ways programming might be strengthened in the future. Firstly, there is a need to improve the conceptualisation of community interventions. Based on the literature, we propose an approach which spans the spectrum of community actions with progressively increasing levels of agency and power (CICICE). At the simplest level, communities are the passive recipients of information; ‘informing’. More complex and multifaceted interventions lead to ‘empowering’. Policymakers and practitioners need to be clearer in specifying what they are trying to achieve with community health interventions, particularly concerning pandemic preparedness.

Secondly, there are important areas where more robust evaluation approaches are needed to help us understand whether CICICE interventions are truly helping to bring about change and delivering value for money. For example, despite the centrality of RCCE to prevention and control work for COVID-19, we found very little peer-reviewed or grey literature on risk communication approaches, and much of what was available was of low quality. This finding is in keeping with those of other reviews on this topic [65]. While a large body of grey literature on risk communication exists, this is not collated in a systematic way which means that key findings are still largely inaccessible to decision-makers. Identifying ways to synthesise this evidence to inform actionable recommendations will be important for future preparedness and should be a priority for researchers.

Thirdly, closer attention is needed in research design, especially given the complex causal pathways from community-level interventions to preparedness. Partly, this is about the composition of research teams: work on HIV/AIDS and Ebola more frequently involved interdisciplinary collaborations between public health specialists and social scientists than for COVID-19. This influenced the ways in which research questions were framed, and the methods applied. However, there was also a question of research design. Much of the evaluation work we considered was observational or quasi-experimental in design. While a proper understanding of the role of context in shaping intervention effectiveness is vital, the growing use of innovative approaches to the evaluation of multi-component community engagement and empowerment interventions in HIV [e.g. 18, 34, 36] shows that there is scope to use these designs more broadly to help us understand what works for pandemic preparedness. There is much that those working on COVID-19 and other diseases of epidemic potential might learn from this.

Future research work should also address a series of additional areas to support strengthened translation of robust CICICE approaches. Firstly, operational aspects of intervention design and delivery need clarification. Many included studies did not detail operational aspects (e.g., staffing requirements, training needs, other resource needs to support delivery). Secondly, a better understanding is needed of the cost implications of different intervention approaches, especially for resource-constrained settings. Finally, the strength of collaboration between social science and epidemiological researchers in HIV was notably greater than for many of the other diseases considered in this review. Forging interdisciplinary collaborations like these will be important in supporting the generation of evidence to inform preparedness in future.

## Conclusions

Putting communities front and centre in epidemic and pandemic preparedness for the future and ensuring the implementation of evidence-based CICICE approaches will be vital for equitable and impactful responses. To better inform CICICE activities for epidemic and pandemic preparedness, future research work should clarify the scope of community and engagement (and related terms) being applied, strengthen methods for evaluating intervention effects, and review the composition of research teams. Teams should incorporate cross-disciplinary perspectives and community members from project inception through to completion and beyond to support co-production and ensure meaningful improvements in preparedness on the ground.

## Supporting information

S1 Checklist PRISMA-SCR checklist for the study. (DOCX)

S1 Text Search strategy. (DOCX)

S1 Table Conceptually oriented studies and grey literature included in the review, with summaries of key findings from these. (DOCX)
