# Correction: Community interventions for pandemic preparedness: A scoping review of pandemic preparedness lessons from HIV, COVID-19, and other public health emergencies of international concern

**DOI:** 10.1371/journal.pgph.0003577

**Published:** 2024-07-25

**Authors:** Sali Hafez, Sharif A. Ismail, Zandile Zibwowa, Nadin Alhamshary, Reem Elsayed, Mandeep Dhaliwal, Fiona Samuels, Ade Fakoya

The images for Figs 2 and 5 are incorrectly switched. The image labelled as Fig 2 should be Fig 5, and the image labelled as Fig 5 should be Fig 2. Additionally, the images for Figs 3 and 4 are also switched. The image labelled as Fig 3 should be Fig 4, and the image labelled as Fig 4 should be Fig 3. The figure captions are in the correct order.

Please see all the correct figures and captions here.

**Fig 1:**
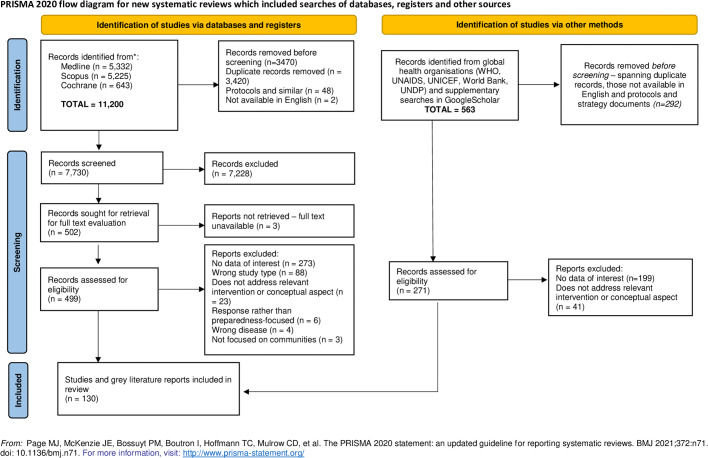
PRISMA flowchart describing the article screening process.

**Fig 2:**
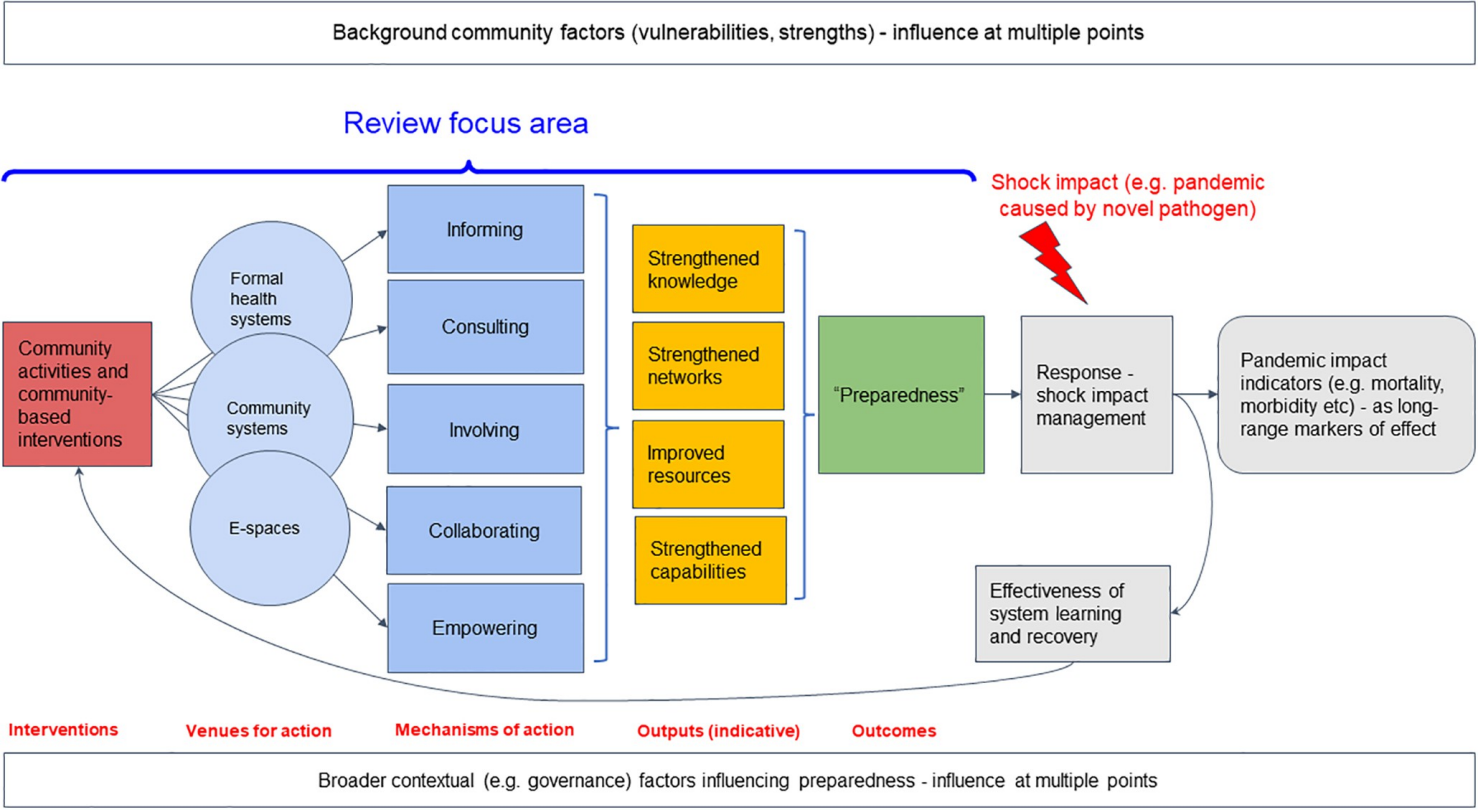
The working theory of change for CICICE activities informing this analysis. The review focuses on measures contributing to directly improved preparedness in anticipation of epidemics and pandemics. However, we acknowledge the importance of feedback and learning (represented here by a feedback loop) from response work.

**Fig 3:**
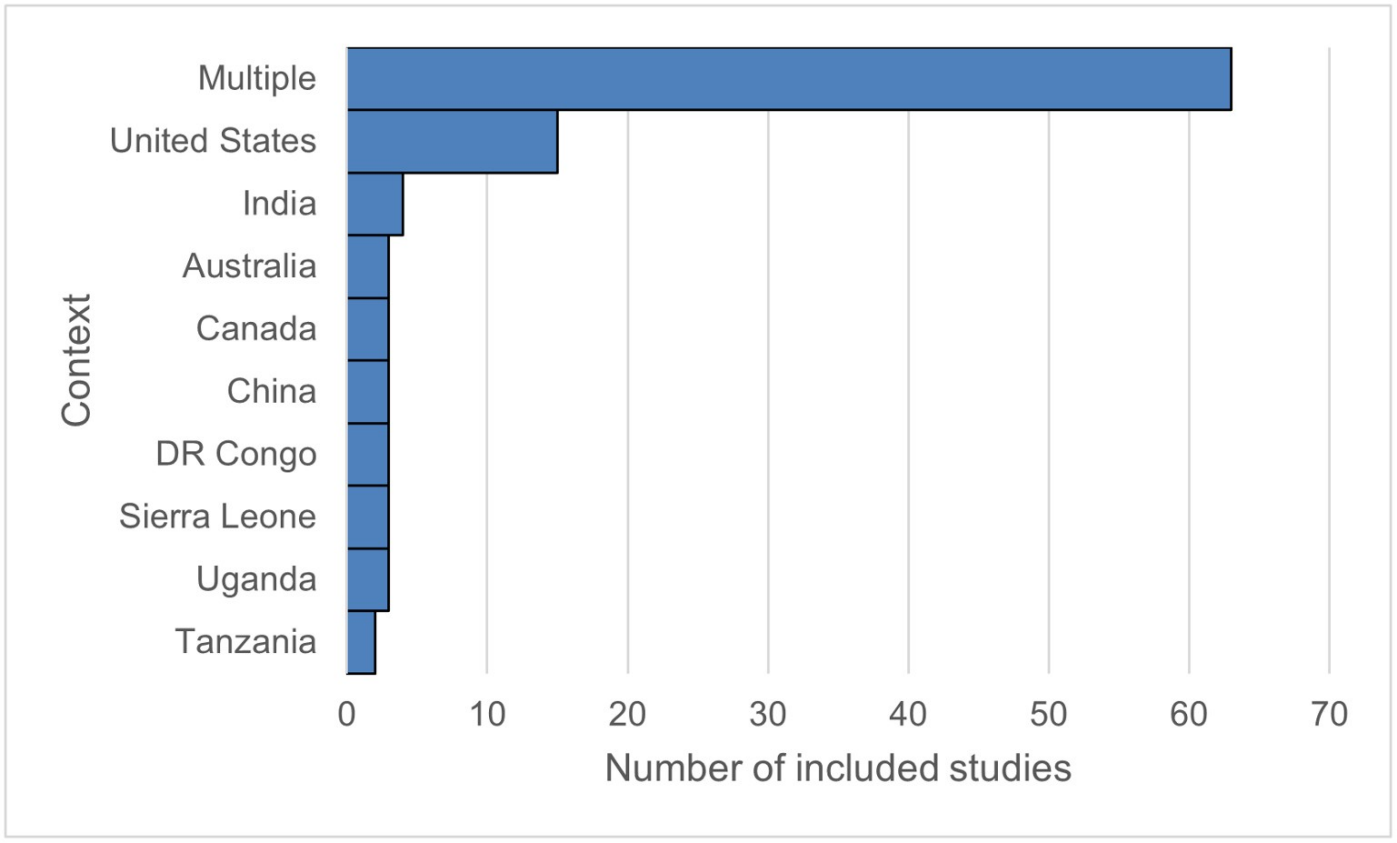
Distribution of studies included by country in which the analysis was set. Those labelled “multiple” included reviews (systematic and narrative), comparative studies, and guidance documents issued by selected global health organisations.

**Fig 4:**
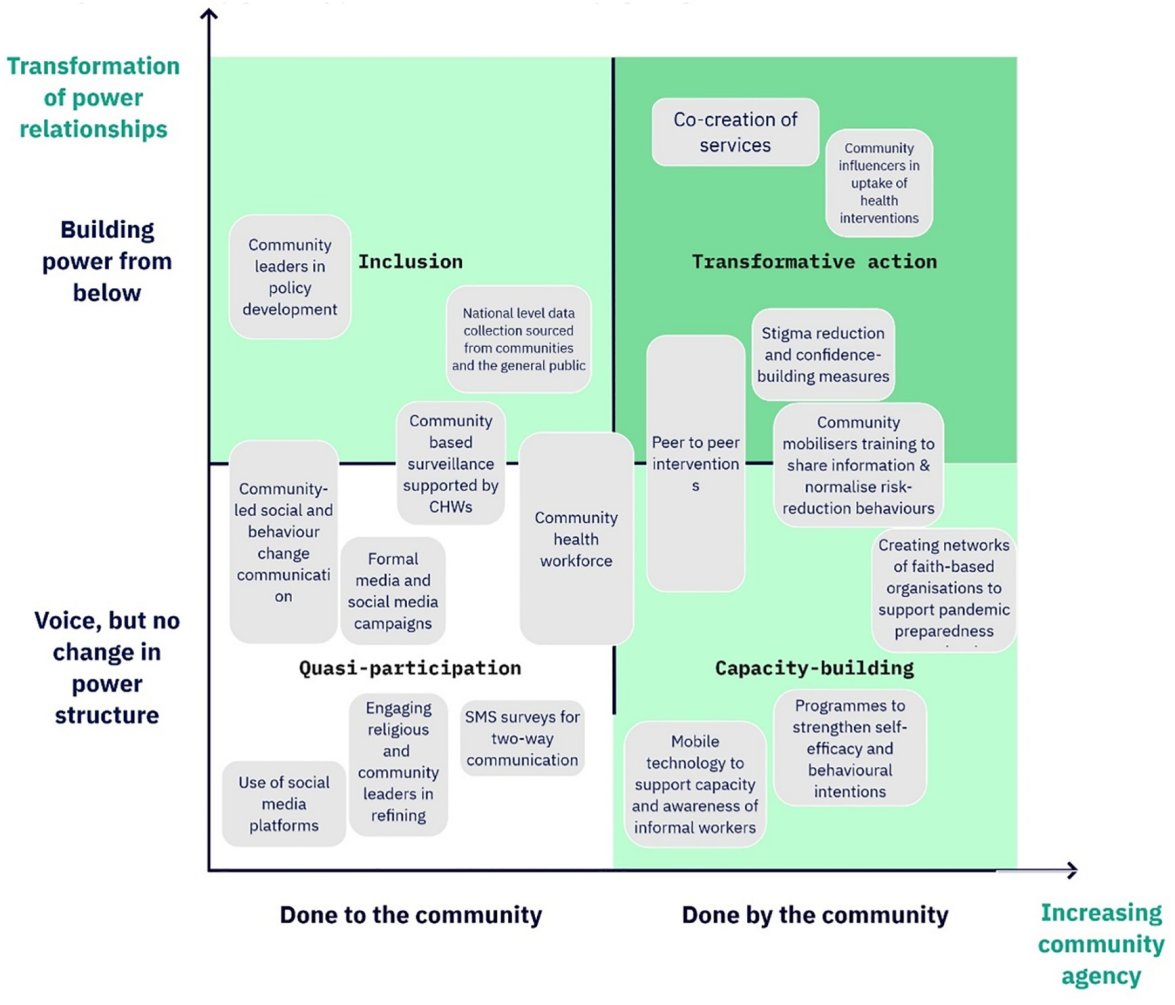
Mapping the diversity of literature on interventions within the realm of CICICE interventions for epidemic preparedness along two dimensions: The extent to which communities develop true agency (x-axis); and the degree to which underlying power relations are transformed (y-axis). Titles in the four quadrants identified how far along a pathway to transformation each form of intervention lies. In this visualisation, individual studies are mapping according to where they fall; systematic reviews and scoping reviews are necessarily excluded because they typically included interventions of many different types (Adapted from [39]).

**Fig 5:**
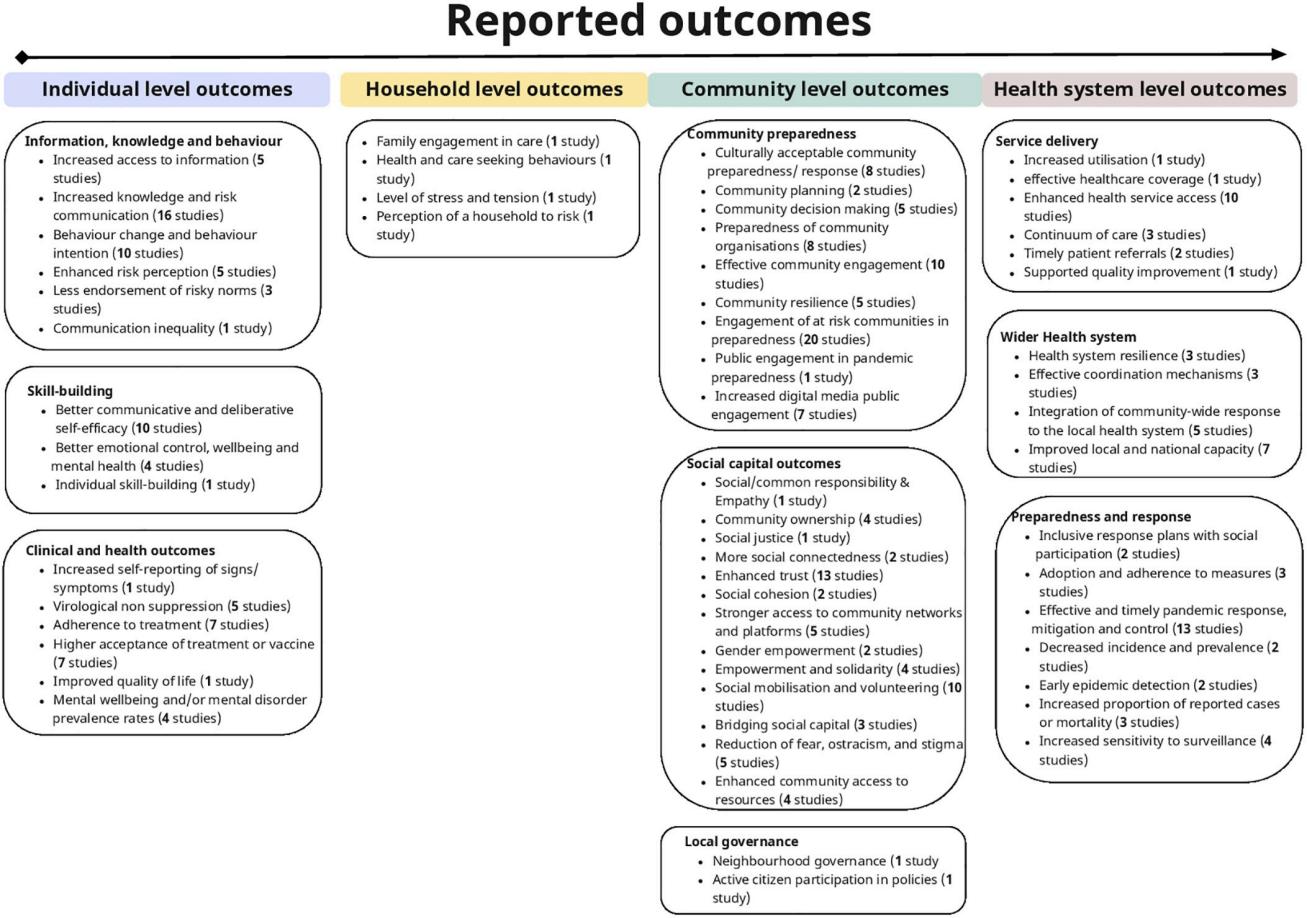
The spectrum of outcomes reported in studies included in the review, ranging from those framed at individual level, through households to communities and finally health system level. Included studies frequently addressed more than one of these outcomes concurrently, though the intervention described was framed at community level (to meet the inclusion criteria for the review). Numbers of studies addressing each outcome are given in brackets.
