# Supplementary material for: Community interventions for pandemic preparedness: A scoping review of pandemic preparedness lessons from HIV, COVID-19, and other public health emergencies of international concern
Source: PLOS Glob Public Health. 2024 May 6;4(5):e0002758. doi: 10.1371/journal.pgph.0002758 (PMC11073720; doi:10.1371/journal.pgph.0002758)
Supplement: S1 Text — (DOCX) [file pgph.0002758.s002.docx]

### S1 TEXT: Search strategy

The search approach for this review was structured using the criteria set out in Table 2 (modified PICO):

| ***Domain*** | ***Criteria*** |
| --- | --- |
| Population | “Communities” understood as being distinct from health service providers (not prescriptive about the definition since a function of the review is to explore the range of definitions applied) |
| Intervention | Any community engagement intervention – see terms below |
| Comparator | Likely to vary according to the study |
| Outcome | Preparedness, recovery, resilience, capacity, empowerment |
| Disease coverage | Ebola, influenzas of pandemic potential (including H1N1 and H5N1), HIV/AIDS, Middle East Respiratory virus syndrome (MERS), MPOX, SARS-CoV1, SARS-CoV2, Zika |
| Sources | Peer reviewed literature databases: MEDLINE, Cochrane Central, Scopus  Grey literature: keyword structured searches of websites for key organisations – WHO, UNICEF, UNAIDS, the World Bank, Independent Panel for Pandemic Preparedness and Response, Global Preparedness Monitoring Board |
| Search dates | 01/01/2008-date of the search for PHEICS and COVID-19  01/01/2000-date of the search for HIV/AIDS |
| Study types | - Journal articles, conference abstracts, agency and NGO evaluation reports - For peer-reviewed papers, study designs may span systematic reviews and meta-analyses, randomised controlled trials, interrupted time series analyses, repeated cross-sectional studies (i.e. primary studies), and narrative (conceptual reviews). Commentaries/editorials, letters, and protocols were not included. - Grey literature sources will need to report original findings from population-based studies of the kinds identified above, or programme or policy evaluations. |
| Geographical focus | Global – no geographical restrictions |
| Languages | English |

**Table A. Summary of the search criteria applied in the review**

A sample search strategy – in this case as applied in the Cochrane database – is set out below as an example of how the search criteria were applied in practice.

| ID Search Hits  #1 communit* OR public OR social OR (civil societ*)  #2 action OR consultat* OR engage* OR involve* OR mobile$* OR participat* OR Plan* OR partner*  #3 #1 AND #2  #4 MeSH descriptor: [Community Participation] this term only  #5 MeSH descriptor: [Community Networks] explode all trees  #6 MeSH descriptor: [Community Support] explode all trees  #7 MeSH descriptor: [Community Resources] explode all trees  #8 MeSH descriptor: [Community Health Planning] explode all trees  #9 MeSH descriptor: [Capacity Building] explode all trees  #10 MeSH descriptor: [Social Participation] explode all trees  #11 MeSH descriptor: [Stakeholder Participation] explode all trees  #12 #4 OR #5 OR #6 OR #7 OR #8 OR #9 OR #10 OR #11  #13 #3 OR #12  #14 PHEIC* OR SARS OR SARS$CoV$1 OR HIV OR AIDS OR MERS OR MERS$COV OR Zika OR Ebola OR (Bird flu) OR (Swine flu) OR (Pandemic Influenza) OR (Influenza A) OR H5N1 OR H1N1 OR COVID$19 OR Conoravirus* OR SARS$COV$2 OR monkeypox OR Mpox  #15 MeSH descriptor: [Disease Outbreaks] explode all trees  #16 MeSH descriptor: [Epidemics] explode all trees  #17 MeSH descriptor: [Pandemics] explode all trees  #18 MeSH descriptor: [Severe acute respiratory syndrome-related coronavirus] explode all trees  #19 MeSH descriptor: [SARS-CoV-2] explode all trees  #20 MeSH descriptor: [COVID-19] explode all trees  #21 MeSH descriptor: [Middle East Respiratory Syndrome Coronavirus] explode all trees  #22 MeSH descriptor: [Coronavirus Infections] explode all trees  #23 MeSH descriptor: [Zika Virus Infection] explode all trees  #24 MeSH descriptor: [Zika Virus] explode all trees  #25 MeSH descriptor: [Influenza, Human] explode all trees  #26 MeSH descriptor: [Monkeypox] explode all trees  #27 MeSH descriptor: [Monkeypox virus] explode all trees  #28 MeSH descriptor: [HIV] explode all trees  #29 MeSH descriptor: [Acquired Immunodeficiency Syndrome] explode all trees  #30 #15 OR #16 OR #17 OR #18 OR #19 OR #20 OR #21 OR #22 OR #23 OR #24 OR #25 OR #26 OR #27 OR #28 OR #29  #31 #14 OR #30  #32 Preparedness OR Recovery OR resilience OR capacity* OR empowerment  #33 MeSH descriptor: [Social Cohesion] explode all trees  #34 MeSH descriptor: [Empowerment] explode all trees  #35 #33 OR #34  #36 #32 OR #35  #37 #13 AND #31 AND #36 with Cochrane Library publication date Between Jan 2008 and Mar 2023 |
| --- |

**Box A. The Sample Search Strategy**
