# Supplementary material for: Community interventions for pandemic preparedness: A scoping review of pandemic preparedness lessons from HIV, COVID-19, and other public health emergencies of international concern
Source: PLOS Glob Public Health. 2024 May 6;4(5):e0002758. doi: 10.1371/journal.pgph.0002758 (PMC11073720; doi:10.1371/journal.pgph.0002758)
Supplement: S1 Table — (DOCX) [file pgph.0002758.s003.docx]

### S1 Table: Table of included studies

**Table A. Conceptually-oriented studies and grey literature included in the review, with summaries of key findings from these**

| ***Authors*** | ***Year*** | ***Study design*** | ***Disease*** | ***Setting*** | ***Community intervention terms used and definitions applied*** | ***Findings*** |
| --- | --- | --- | --- | --- | --- | --- |
| Alberti et al (152) | 2020 | Narrative review | COVID-19 | United States | Community engagement = "the application of institutional resources (e.g., knowledge and expertise of faculty and students, technical infrastructure, and physical space) to address and solve challenges facing communities through collaboration with those communities" | Meaningful community engagement will be central to ensuring equitable preparedness and response to future pandemics. Communities need to be at the centre of decision-making around resource deployment to ensure the health and wellbeing of all. |
| Coleman et al (153) | 2012 | Narrative review | HIV | United States | Not defined | The study offers a framework for implementing HIV/AIDS prevention programs in African American churches to provide locally developed, culturally appropriate HIV/AIDS prevention programs. Having dedicated individuals with previous HIV/AIDS experience and the desire to help their church and community were essential to the implementation of faith-based HIV/AIDS prevention programs. |
| DeBruin et al (154) | 2012 | Commentary | Multiple | United States | Not defined | Pandemic planning should be informed by a social justice approach, with a focus on partnering with at-risk communities and attention to resource distribution to ensure that vulnerable communities are not disproportionately affected by future pandemics. |
| Dickmann et al (52) | 2015 | Commentary | Multiple | Multiple | Risk communication = an inter-active, holistic, continuous and engaging activity that focuses on dialogue, intelligence gathering, building relationships over time with a knowledge base informed by new and accessible communication technologies (e.g. social media and networks) and supportive environments. | Evaluation of risk communication activities should focus on the extent to which they lead to earlier detection, faster response, smoother coordination and a smarter legacy (ability to use processes and outcome measures to improve current and future performance) as mechanisms for improving response capacities . |
| Gilmore et al (155) | 2020 | Rapid evidence synthesis | Multiple | Multiple | Community engagement = “involvement and participation of individuals, groups and structures within a parameter of a social boundary or catchment area of a community for decision-making, planning, design, governance and delivery of services” | Engagement can occur on a spectrum from passive forms (provision of information) to active forms (e.g. collaboration involving shared decision-making). Most evidence addresses passive approaches; there are few data on equity-building approaches. Many different types of actor are important in supporting engagement, including local leaders, community organisations and faith-based groups and individuals. |
| ILO (156) | 2020 | Guidance document | HIV | Multiple | Not defined | Successfully addressing stigma in epidemic and pandemic preparedness and response depends on a composite approach incorporating rights-based response work, care and attention to use of language, respect for confidentiality, protection of livelihoods, and sharing positive stories – among other strategies. |
| Jewett et al (157) | 2021 | Scoping review | COVID-19 | Multiple | Community engagement = "activities that build trust between all levels of government and its constituents" Social cohersion = "the degree of social connectedness and solidarity between different community groups within a society, as well as the level of trust and connectedness between individuals and across community groups" | Investment (financial, material and in other forms) in community cohesion and resilience are important for post-disaster recovery and future preparedness. Protocols and plans should take into account perspectives from different communities and do so with an equity lens to reduce the risk of marginalisation and inequalities in impact. |
| Kamaka et al (158) | 2021 | Mixed-methods study | COVID-19 | United States | Not defined | Developing culturally-informed frameworks to help ground policy development and practice with respect to indigenous communities is important for improving pandemic preparedness and response, and ensuring that these communities have voice, and equitable approaches to promoting wellbeing are implemented. |
| Khan et al (159) | 2022 | Narrative review | COVID-19 | Multiple | Not defined | To be effective, risk communication efforts must find a middle ground between inadequate approaches (which promote rumours, confusion and fear) and infodemic approaches (where people are overwhelmed by risk information from multiple stakeholders, again promoting fear, or even anger and violence). |
| Liu et al (160) | 2021 | Cross-sectional survey | COVID-19 | China | Not defined | Lack of community participation was associated with lower perceived effectiveness of neighbourhood collaboration in response to COVID-19 (beta=-0.353, p<0.05), whereas perceived prioritisation locally (0.851, p<0.001) or the level of government support (0.307, p<0.01) were positively associated. |
| Lohiniva et al (161) | 2022 | Cross-sectional survey | COVID-19 | Finland | Not defined | Pandemic risk perception is influenced by multiple factors including knowledge, personal experience, trust, cultural values and others. Sociocultural factors including culture (at community level or nationally) and approaches to individual rights are also important. |
| Lok Swasthya SEWA Trust and WHO India (31) | 2021 | Narrative review | COVID-19 | India | Not defined | In India, capacity building of grassroots-level women workers as master trainers was effective in RCCE and opens avenues for performing other tasks such as counselling. The report highlights the acceptance and posistive use of technologies such as mobile facilitation communication channels and platforms for online learning are percieved to be effective in training women leaders, RCCE and service referals. |
| Maunula (162) | 2013 | Narrative review | PHEIC | Canada | Not defined | Current pandemic plans in Canada assume a primary focus on individuals and families (rather than communities) and privilege scientific and medical expert knowledge above other forms of knowing. Alternative formulations should address the role of communities, and consider distinctions between spaces of personal, community and wider societal action, and the role of the state with respect to these. |
| McCollum et al (163) | 2022 | Qualitative study | COVID-19 | Multiple | Not defined | Building trust with local communities is central to effective response - consistent engagement via trusted leaders is a key principle for preparedness. |
| Murthy et al (164) | 2021 | Commentary | Multiple | Multiple | Not defined | A "3 Rs" approach to social media engagement can improve uptake and effectiveness of information for pandemic preparedness purposes: reviewing the target audience; recognising distinct health communication needs; and responding with tailored messages. |
| O'Grady et al (165) | 2022 | Narrative review | Multiple | Multiple | Not defined | An emphasis on community is is important for recognising and integrating formal and informal practices that have developed to mitigate pandemic effects (in the context of COVID-19). The complex relationship between community and government is constantly shifting and is likely to be reshaped by the particular circumstances and trajectories of any future pandemic - influencing the way in which community resilience may be understood. |
| Oliveira et al (166) | 2022 | Narrative review | COVID-19 | Brazil | Not defined | Strengthened trust, solidarity, partnerships, intersectoral action, social mobilization and protection of access to resources for the most vulnerable groups are all pre-requisites for effective pandemic preparedness and response. Strengthening community capital to promote communication and networks for donations is also an important component of this. |
| O'Sullivan and Phillips (167) | 2019 | Commentary | Multiple | Canada | Not defined | New approaches to consultation and engagement are needed for vulnerable communities in pandemic preparedness and response, to ensure community assets (including social networks) are strengthened and to ensure that preparedness and response strategies respond to changing community relations, in a whole-of-society approach. |
| Quinn (168) | 2008 | Narrative review | Multiple | Multiple | Not defined | Effective engagement of racial and ethnic minority communities in pandemic preparedness depends on prior work to build trust and collaboration with partners including community-based organisations, faith communities, civil rights groups and others. Recognised crisis and emergency risk communication (CERC) measures can support this. |
| Rela et al (169) | 2022 | Narrative review | COVID-19 | Indonesia | Community participation = "the participation of community members in developmental programs" | Community engagement and empowerment for pandemic preparedness and response relies on a combination of measures spanning social capital building, communication, the use of inclusive communication technologies, and economic and financial empowerment at individual and household level. |
| WHO (22) | 2020 | Narrative review | Multiple | Multiple | Community engagement = "a process of developing relationships that enable stakeholders to work together to address health-related issues and promote well-being to achieve positive health impact and outcomes" | Principles underpinning community engagement include trust, accessibility, equity, transparency and autonomy. Community engagement can be divided into four levels according to intent – community-oriented at the minimal end, through to community-owned at the maximal end. The latter involves formal mobilisation of community assets and the empowerment of community members to self-govern. |
| WHO (51) | 2017 | Commentary | Multiple | Multiple | Community engagement = "a process of developing relationships that enable stakeholders to work together to address health-related issues and promote well-being to achieve positive health impact and outcomes." | A novel conceptual framework (CEQ) can help frame the contributions of community health workers and others to community engagement – centring engagement as “core business” for all health services and programmes. The CEQ framework distinguishes enabling conditions (e.g. pre-requisites such as governance, peace and democracy; leadership, values and shared vision); capacity development (through situation analysis and context-specific approaches) through to systems and health outcome changes achieved. |
| WHO Europe (170) | 2019 | Commentary | Multiple | Multiple | Not defined | To strengthen the use of social science interventions in pandemic preparedness, WHO trained 24 participants from 11 countries in Europe on SocialNET, which has been implemented in the context of the Pandemic Influenza Preparedness (PIP) Framework, to ensure effective RCCE in outbreaks and health emergencies. |

**Table S3b. Empirically-oriented studies included in the review and summaries of key findings from these studies.**

| ***Authors*** | ***Year*** | ***Study design*** | ***Broad disease focus*** | ***Setting*** | ***Community intervention terms used and definitions applied*** | ***Intervention(s)*** | ***Results*** |
| --- | --- | --- | --- | --- | --- | --- | --- |
| Abdalla et al (63) | 2021 | Narrative review | COVID-19 | Multiple | Community = "groups with a common set of social relationships that formulate a shared identity among members" | Multiple, ranging from use of social media for communication, digital tools for contact tracing through to legislation against online misinformation and encouraging wider cultures of civic mindedness. | Communities should be centred in preparedness and response and should have ownership in all aspects of this from planning through to implementation and evaluation. Effective risk communication depends on timely and accurate information provision using multiple channels and via trusted community leaders. |
| Aung et al (64) | 2021 | Mixed-methods study | COVID-19 | Multiple | Not defined | Multiple, spanning risk communication via different modalities, community outreach work, the use of health volunteers to engage communities and other approaches. | Community health workers played important roles in delivery of PPE and other essential materials to support service delivery, supporting risk communication and helping to ensure food and economic security for those in need. |
| Brewer et al (65) | 2020 | Mixed-methods study | COVID-19 | United States | Not defined | Use of trusted community messengers to support dissemination of information via multiple channels | Community-based participatory research partnerships can be repurposed to support emergency preparedness and risk communication in marginalised communities in pandemics - in this case including social media message dissemination via a network of 120 churches and their congegrations serving African American populations in Minnesota. |
| Choukou et al (66) | 2022 | Scoping review | COVID-19 | Multiple | Health literacy = "the degree to which individuals can obtain, process, understand and communicate about health-related information needed to make informed health decisions" | Digital health interventions to support risk communication | There is little literature on digital interventions and platforms for improving health literacy among vulnerable groups. Facilitators identified included effective communication with healthcare professionals to promote digital technologies, barriers included limited access to digital devices. |
| Cruwys et al (67) | 2022 | Quasi-experimental (e.g. time series analysis) | COVID-19 | Australia | Neighbourhood identification = "the degree to which a person derives a sense of subjective self-definition and affiliation from their local placebased community"  Social identification = "encapsulates not just the experientially pleasant aspects of social interaction, but instead is literally the psychological representation of one's membership in a social group...together with the value and emotional significance attached to this." | "Neigbour Days" in Australia: "a campaign that advocates for and empowers people to organise bespoke activities in their local communities that build social cohesion (e.g., having a neighbourhood barbeque/picnic, home visits to elderly neighbours, setting up a WhatsApp group for their street)." | Neighbour Day participation was associated with a stronger sense of neighborhood identification (p<0.001), higher social cohesion (p=0.012), and more interpersonal ties (p<0.001) compared to non-participants. Although increases in psychological distress and declines in self-reported wellbeing following COVID-19 lockdown imposition were reported across all groups, Neighbour Day participation moderated these effects by comparison with the control group. |
| den Broeder et al (68) | 2022 | Narrative review | COVID-19 | Multiple | Community engagement = "a change process…that provides access to and for vulnerable groups and helps build insight into the assets and needs of residents" | Three-level focus: (i) community/citizen-led responses - mutual aid, informal volunteering and community organising examples; (ii) actions linking the work of organisations with social action by citizens; and (iii) organisation-led actions - initiated by public sector/voluntary sector and coordinated through formal platforms | Six actions - increased mutual aid, centring community-based organisations, promoting volunteering, deploying social media and engaging in novel health promotion activities - are all key elements in ensuring communities are resilient to future pandemics. |
| Fransen et al (69) | 2022 | Narrative review | COVID-19 | Multiple | Not defined | Community resilience initiatives | Community resilience initiatives varied in size and often formed temporarily to address local vulnerabilities highlighted by the pandemic. Availability of funding and materials, and the strength of partnerships and local networks influenced CRI formation and effectiveness. Drivers for emergence were multi-level and not solely bottom-up - so attention is needed to the role of a broad range of stakeholders in supporting CRI development. |
| Junior and Morais (47) | 2020 | Narrative review | COVID-19 | Brazil | Various definitions of "community" described: The most widespread is geographically-defined; other approaches inc those based on social and economic characteristics, or values and traditions. | Empowerment through health councils involving community members in decision-making | Health Councils are effective tools for ensuring both citizen education (on e.g. health risks) but also engaging participation in decision-making, provided equitable composition, a meaningful role in decision-making and responsibility for health financing as well as service delivery. |
| Kalocsányiová et al (70) | 2022 | Scoping review | COVID-19 | Multiple | Not defined | Multiple but with a primary focus on RCCE | Multiple inequalities in messaging deployment and effects were identified by race, language, ethnicity, gender, age, geographical location and underlying health status. Barriers for different populations included language, insufficient attention to lived experience and the specific circumstances of different communities, and ineffective communication channels. The impact of communication interventions is often poorly evaluated. |
| Lau (71) | 2020 | Narrative review | COVID-19 | China | Not defined | Ground-up social mobilisation | Community-level social mobilisation, often along lines of political affiliation, had an important role in supporting preparedness and early response to COVID-19 in Hong Kong. This relied in part on networks and forms of social capital built up during periods of political protest preceding the pandemic. |
| Lim and Nakazato (72) | 2020 | Cohort study | COVID-19 | Japan | Not defined | Ground-up social mobilisation | Network dynamics influence uptake of risk communication information. Students in this study were more likely to adopt voluntary public health measures if they knew other students who had done so (p<0.05), and tended to seek out information from others with subjective health statuses markedly different from their own. |
| Maher and Murphet (73) | 2020 | Narrative review | COVID-19 | Australia | Not defined | Risk communication | Circulating and reinforcing key information via trusted and informal, two-way communication networks are important in ensuring the effectiveness of risk communication efforts and that community buy-in is strong. Other factors including language and literacy, interpersonal relationships, which sources people regard as trustworthy and the nature and extent of engagement with social media and other platforms are also important. |
| Mahmud et al (74) | 2021 | Mixed-methods study | COVID-19 | Bangladesh | Not defined | Community based communicable disease surveillance | Over the 10 weeks of the study, the self-reported syndromic surveillance system received approximately 3.5 million responses. Data trends align with confirmed cases of COVID-19 over the same time period with a lag time of one to two weeks, including case rates among people classified as vulnerable to severe disease. Self-reported data provide an early indication of outbreak spread. Participatory surveillance on this model can complement other surveillance systems. |
| Malik et al (75) | 2021 | Mixed-methods study | COVID-19 | Multiple | Not defined | Risk communication via social media | Instagram may be a useful tool for engaging younger audiences in particular in risk communication activities (based on limited data on user engagement, featuring total number of views and mean number of comments/likes). |
| Mat Dawi et al (76) | 2021 | Cross-sectional survey | COVID-19 | Malaysia | Not defined | Risk communication | Use of e-government tools (beta= 0.205, p < 0.01) and social media (beta = 0.081, p < 0.05) were statistically significant predictors of attitude toward protective behavior engagement against COVID-19 in this population. |
| Nöstlinger et al (77) | 2022 | Narrative review | COVID-19 | Belgium | Not defined | Risk communication and community outreach | Use of translated and tailored information, and direct outreach to community members with complex needs (e.g. elderly, those with irregular legal status or small social networks) were identified as effective strategies for community engagement to maximise adherence to/uptake of community control measures for COVID-19. |
| Rämgård et al (78) | 2023 | Qualitative study | COVID-19 | Sweden | Not defined | Use of "lay health promoters" to support trust-building at community level | The existing structure of a community-based participatory research programme, alongside lay health promoters to build trust, could help disseminate relevant risk information and ensure that marginalised community members are engaged in building the resilience and sustainability of their communities to future pandemics, including by performing a bridging function with local authorities, and supporting dialogue. |
| Rashmi and Lekshmi (79) | 2021 | Commentary | COVID-19 | India | Not defined | Multiple | Multiple modes of social mobilisation supported pandemic preparedness and response to COVID-19 in Kerala, including small scale activities such as community kitchens, the use of community-based surveillance, and community-based production of PPE (facemasks). |
| Rezaei et al (80) | 2022 | Cross-sectional survey | COVID-19 | Iran | Not defined | Capacity building through community based organisations | Preparedness of community based organisations relies on planning, training, infrastructure of these organisations. Organisations prioritised resource allocation and the medical needs of clients. |
| Sahoo et al (44) | 2023 | Scoping review | COVID-19 | Multiple | Community engagement and involvement = "participation of individuals, groups, and structures within the social boundary in decision-making, planning, design, governance, and service delivery" | Multiple including community based disease surveillance, use of CHWs and others | Specific community volunteers, community-based organizations, and civil society organizations had high interest but less influence, indicating that it is necessary to recognize and engage them. Motivation is crucial for those with high influence but less interest, such as corporate responsibility for the health system’s preparedness plan among urban populations. |
| Wild et al (81) | 2021 | Narrative review | COVID-19 | Australia | Not defined | Risk communication and community outreach | Effective health communication for COVID-19 pandemic requires building partnerships between culturally and linguistically diverse leaders, communities and government. This partnership can be materialised in disseminating messages, designing messages and building trust between communities and government, putting the the diverse needs and circumstances of people and communities at the centre of health communication and behaviour change strategies. |
| Tambo et al (82) | 2021 | Narrative review | COVID-19 | Multiple | Not defined | Risk communication and community engagement | In China, an effective RCCE strategy involves the national government and political leaders, working together with public health experts, by establishing COVID-19 risk commnication system using whole-of-government approach, enhanced local partnership and leadership, strengthening public trust and participatory risk communication, improved community engagement and resilience, and re-enforcing pandemic capacity building and community health workers competencies at all levels. |
| WHO (83) | 2022 | Guidance document | COVID-19 | Multiple | Community engagement = "a process of developing relationships that enable stakeholders to work together to address health-related issues and promote well-being to achieve positive health impact and outcomes" | Risk communication | Implementation guidance document focused on infodemic management in RCCE in Europe. Results not described. |
| WHO (84) | 2022 | Guidance document | COVID-19 | Multiple | Not defined | Multiple, but a strong focus on community representation through recognised community leaders or representatives of specific vulnerable groups | The report collates best practices on community assets and civil society outreach and how they supported health systems and community resilience in COVID-19 pandemic, spanning from protecting healthcare workers from attacks in Burkina Faso, working with young people in Kisumu and Nairobi in Kenya on RCCE and preventive measures, engagement with indigenous communities in Ecuador, getting persons with disabilities involved in health emergencies in Guatemala. |
| IFRC, UNICEF, WHO (85) | 2020 | Guidance document | COVID-19 | Multiple | Not defined | Community based healthcare delivery including by community health workers | Interim guidance document on community based healthcare, including outreach and campaigning, in the COVID-19 pandemic. Results not described. |
| UNICEF, WHO (86) | 2021 | Guidance document | COVID-19 | Multiple | Not defined | Risk communication and community outreach | Interim guidance on community engagement for COVID-19 vaccines. No reported results |
| IFRC, UNICEF (87) | 2020 | Guidance document | COVID-19 | Multiple | Not defined | Focus groups as a community consultation and engagement technique | Guidance on conducting focus groups discussions with the community, as part of the risk communication and community engagement for COVID-19. No reported results. |
| IFRC, UNICEF, WHO (88) | 2020 | Guidance document | COVID-19 | Multiple | Not defined | Risk communication and community outreach | Guidance for community workers, volunteers and community networks on engaging with the communities for the COVID-19 pandemic, focusing on how to engage, asking the right questions and what to say. No reported results. |
| GOARN, IFRC, UNICEF, WHO (89) | 2021 | Guidance document | COVID-19 | Multiple | Community engagement = "an approach to directly involve local populations in all aspects of decision-making implementation, and policy. Building on a participatory approach, community engagement strengthens local capac- ities, community structures and local ownership to improve transparency, accountability and optimal resource allocations across diverse settings." | Community based contact tracing | Operational guide for engaging communities in contact tracing. No reported results. |
| IFRC, UNICEF, WHO EMRO (59) | 2020 | Guidance document | COVID-19 | Multiple | Community engagement = "a process of developing relationships that enable people of a community and organizations to work together to address health-related issues and promote well-being to achieve positive health impact and outcomes." | Risk communication and community engagement | Regional guiding framework for risk communication and community engagement for the COVID-19 response in the Eastern Mediterranean Region/Middle East and North Africa. No reported results. |
| WHO Europe (90) | 2021 | Guidance document | COVID-19 | Multiple | Not defined | Risk communication and community engagement | Interim guidance document on risk communication and community engagement for COVID-19 contract tracing. No reported results. |
| UNICEF, WHO Europe (91) | 2022 | Guidance document | COVID-19 | Multiple | Not defined | Risk communication and community engagement | Implementation tool focusing on risk communication and community engagement for COVID-19 vaccination. No reported results. |
| WHO (92) | 2020 | Guidance document | COVID-19 | Multiple | Not defined | Risk communication and community engagement | Interim guidance on risk communication and community engagement readiness and initial response for novel coronaviruses (nCoV). No reported results. |
| WHO (93) | 2020 | Guidance document | COVID-19 | Multiple | Not defined | Risk communication and community engagement | Interim guidance on risk communication and community engagement readiness and response to coronavirus disease (COVID-19). No reported results. |
| WHO Europe (94) | 2022 | Guidance document | COVID-19 | Multiple | Not defined | Risk communication and community engagement | The report captures a list of lessons learned from implementing RCCE interventions in times of COVID-19 through a series of case studies, spanning from the legal duty to communicate with a whole-of-government approach in Finland, the role of RCCE preparedness in supporting transparent and timely communication in Bosnia and Herzegovina, Azerbaijan's 24/7 COVID-19 hotline role in addressing public concerns, and similar actions. |
| WHO Western Pacific Region (95) | 2020 | Guidance document | COVID-19 | Multiple | Not defined | Risk communication and community engagement | Interim guidance on the role of community engagement in situations of extensive community transmission of COVID-19. No Reported results. |
| UNDP (96) | 2020 | Guidance document | COVID-19 | Bosnia and Herzegovina | Not defined | Multiple, including engagement with recognised community leaders | The report explains the level of preparedness of local structures, knowledge and suggested actions for different COVID-19 measures, in communication, preparedness action, public engagement, introducing new services to the public, however, it does not measure the effectiveness of these preparedness measures |
| Abramsky et al (112) | 2014 | Randomised controlled trial | HIV | Uganda | Not defined | Community mobilisation through network building, peer-to-peer work and other measures | A community mobilization program was associated with significantly lower social acceptance of IPV among women (adjusted risk ratio 0.54, 95% confidence interval (CI) 0.38 to 0.79) and lower acceptance among men (0.13, 95% CI 0.01 to 1.15); significantly greater acceptance that a woman can refuse sex among women (1.28, 95% CI 1.07 to 1.52) and men (1.31, 95% CI 1.00 to 1.70); 52% lower past year experience of physical IPV among women (0.48, 95% CI 0.16 to 1.39); and lower levels of past year experience of sexual IPV (0.76, 95% CI 0.33 to 1.72). |
| Akeju et al (98) | 2021 | Qualitative study | HIV | Nigeria | Not defined | Capacity building through community-based organisations | Community-Based Organizations (CBOs) providing HIV prevention services in Nigeria exhibit varied management practices, impacting service efficiency; established CBOs have better infrastructure and planning, while newer ones lack these attributes, indicating that addressing issues like skills, organizational structure, and talent retention can enhance HIV service delivery. |
| Bauman et al (46) | 2021 | Randomised controlled trial | HIV | United States | Not defined | Multiple, spanning risk communication, community engagement and trust-building initiatives, and the development of self-efficacy among an at-risk group | PREPARED interventions (11 session program and a 3-week internship) had significant improvements compared to TEEN communication skills interventions after 6 months in HIV knowledge, sexual self-efficacy, and outcome expectancy for condom use among young people. At one year, there were significant differences favouring PREPARED in outcome expectancy for condom use (TEEN= 34.6, PREPARED= 35.1), sexual self-efficacy (TEEN=63.8, PREPARED=67.3), and intention for partner communication about HIV/AIDS or STIs (TEEN= 40, PREPARED |
| Beattie et al (42) | 2014 | Cross-sectional survey | HIV | India | Empowerment = "the processes by which those who have been denied the ability to make choices (disempowered) acquire such an ability" | Multiple, spanning risk communication, network building and community mobilisation and empowerment principally through peer-to-peer work | Female sex workers with high community mobilisation exposure were more likely to have been tested for HIV (AOR = 25.13) and to have used a condom at last sex with occasional clients (AOR = 4.74), repeat clients (AOR = 4.29), and regular partners (AOR = 2.80) than those with low community mobilisation exposure. |
| Carballo-Dieguez et al (99) | 2005 | Randomised controlled trial | HIV | United States | Empowerment = "collective learning efforts in which individuals gain a critical understanding of the relevant social environment, access to resources, and work with others to achieve common goals and take action to exert control in their lives" | Multiple, but focusing on network building, strengthening mutual understanding and coalition building to promote empowerment | A series of 8 interactive sessions, developed by Latino gay men is found to be effective in HIV behaviour change. At first follow-up, 46% of experiment participants reported no Unprotected Anal Intercourse (UAI) during the prior two months, similar to the control group. At second follow-up, 44% of men in the intervention group, and 40% in the control group reported no UAI, and at FUA3, these values were 51% and 46% respectively. |
| Carbone et al (100) | 2019 | Qualitative study | HIV | Malawi | Not defined | Peer-to-peer intervention | Peer-led, non-judgmental prevention of mothers-to-child transmission of HIV support services are found to be preferred interventions by adolescent mothers in prevention of mother-to-child transmission of HIV. Poverty, stigma, food insecurity, lack of transport, and absence of psychosocial support were crosscutting barriers to mothers engagements, while resilience and self-efficacy were key identified as key motivating factors. |
| Carlson et al (101) | 2012 | Randomised controlled trial | HIV | Tanzania | Empowerment = the combination of social cohesion and the willingness to take civic action, the health benefits of collective efficacy have been shown to impact violence, birth weight, mental health and the age of onset of sexual intercourse | Multiple, but focusing on network building, strengthening mutual understanding and coalition building to promote empowerment | A 28 week Youth Citizen Program on HIV prevention is found to be affected among young people. Deliberative self-efficacy (effect side= 0.27, confidence interval, CI=0.44-1.56), communicative self-efficacy (effect size= 0.30, CI = 0.6-1.77) and emotional control (effect size= 0.17, CI=0.05-0.77) were significantly higher in the treatment group. |
| Choi et al (102) | 2022 | Randomised controlled trial | HIV | Multiple | Not defined | Risk communication and community engagement via an online platform | Digital HIV interventions with personalized intervention components that consider the individuals’ differences could increase their engagement and efficacy. The study found that educated people logged in more (β=.22; P=.045). Also, the number of sessions viewed was negatively associated with changes in internalized homophobia (β=–.06; P<.001) and with changes in perceived usefulness of online dating for hookups (β=–.20; P<.001). |
| Cohen et al (45) | 2022 | Randomised controlled trial | HIV | Kenya | Not defined | Multiple, including provision of an agricultural loan alongside training in sustainable agriculture and financial literacy | The multisectoral agricultural intervention led to demonstrable health and other benefits; however, it was not possible to detect additional effects of the intervention on HIV clinical indicators. HIV viral suppression improved in both groups from baseline to end of follow-up from 314 of 366 (85.8%) to 327 of 344 (95.1%) in the intervention group and from 291 of 353 (82.4%) to 314 of 333 (94.3%) in the control group (P = .86). |
| Dunbar et al (56) | 2020 | Systematic review | HIV | Multiple | Not defined | Multiple, spanning risk communication and community engagement | Three stigma reducing interventions mechanisms highlighted: firstly, by enhancing self-acceptance, leadership, and behaviour change motivation through intrapersonal approaches; secondly, by promoting socialization, knowledge sharing, and empowerment through interpersonal strategies; and thirdly, by fostering community introspection, self-reflection, and humanistic activation through structural strategies. |
| Feyissa et al (103) | 2019 | Systematic review | HIV | Multiple | Not defined | Multiple, including risk communication, identification and support for community leaders, peer-to-peer work | Training popular opinion leaders resulted in significantly lower mean avoidance intent scores (MD = -1.87 [95% CI -2.05 to -1.69]), mean prejudicial attitude scores (MD = -3.77 [95% CI -5.4 to -2.09]) and significantly higher scores in mean compliance to universal precaution (MD = 1.65 [95% CI 1.41 to 1.89]). |
| Gulaid and Kiragu (29) | 2012 | Narrative review | HIV | Multiple | Community engagement = "participation, mobilization, and empowerment while excluding activities that involve communities solely as service recipients" | Multiple, spanning community health workers, network building and trust-building through engagement of community leaders, peer-to-peer work and other approaches. | Promising practices to increase the supply of preventing mother-to-child transmission (PMTCT) services included extending community cadres, strengthening linkages with community, FBOs, and civic participation in program monitoring. Practices to improve demand for PMTCT included community-led social and behaviour change communication, peer support, and participatory local solutions. |
| Haberer et al (97) | 2021 | Narrative review | HIV | Multiple | Not defined | Using socio-behavioural science to strengthen the formulation of community-oriented interventions | Transparent communication, and trust-building and credible engagement approaches were important in shaping individual and community-level behavioural changes during the HIV pandemic and are likely to be key for current and future pandemic responses (including for COVID-19). |
| (58)Harrison (104) | 2019 | Randomised controlled trial | HIV | China | Not defined | Capacity building intervention focused on vulnerable groups | Caregivers training to enhance the psychological wellbeing of children affected by HIV resulted in decreased anxiety and parental stress at 12 months ((β = −0.106, P < 0.05), increased use of structured parenting skills at 12 and 24 months (β = 0.083 and 0.076, respectively), followed by significantly lower levels of parental competence at 36 months. |
| Hickey et al (105) | 2015 | Quasi-experimental (e.g. time series analysis) | HIV | Kenya | Community engagement = the involvement of individuals within a community in efforts to improve long-term engagement in HIV care and medication adherence. | Capacity building intervention to strengthen social capital | A Microclinic social network intervention on community engagement in HIV improve long-term engagement in HIV care and possibly medication adherence. Intervnetion participants experienced one-half the rate of ≥ 90-day clinic absence as those in control communities (adjusted hazard ratio 0.48, 95%CI 0.25–0.92). |
| (106)Kerrigan et al (107) | 2017 | Randomised controlled trial | HIV | Tanzania | Not defined | Multiple, including peer-led education, peer-led care navigation, network building and community mobilisation activities | Being part of a community (OR 4.27), age (OR 7.09), shorter duration of time working at venue (OR 0.21), higher number of clients per week (OR 0.10), substance use during sex work (OR 0.31), and social cohesion (OR 5.33) were associated with HIV viral suppression among female sex workers. |
| Kerrigan et al (57) | 2013 | Systematic review | HIV | Multiple | Community empowerment = "a social action process that promotes the participation of people, organizations, and communities towards the goals of increased individual and community control, political efficacy, improved quality of community life, and social justice" | Multiple, spanning risk communication, peer-led education and community mobilization activities to support empowerment for a vulnerable group | Community empowerment-based HIV prevention was associated with significant improvements across HIV outcomes and settings. For HIV infection, two observational studies showed a significantly protective combined effect [(OR): 0.84, 95 % (CI): 0.709–0.988]. |
| Kiragu et al (43) | 2020 | Systematic review | HIV | Multiple | Community defined by population. Engagement defined in terms of the specific development approach used (see interventions column) | Multiple, including peer-led education and outreach, community mobilisation, organisational capacity development, and socioeconomic empowerment | Ample evidence links capacity development to uptake of services, and dearth of research examining the impact of capacity development on demand for rights among key population. Positive effects from range of community mobilisation and organisational capacity development interventions on condom/lubricant use, and on intermediate outcomes (knowledge, protection self-efficacy etc). |
| Li et al (108) | 2022 | Randomised controlled trial | HIV | Vietnam | Not defined | Multiple, spanning community health workers, network building and trust-building through peer-to-peer work, and risk communication | The community capacity-building intervention had shown promising yet limited adherence outcomes among a subset of People living with HIV who use drugs (PLHWUD) who had already initiated ART. There is an increased motivation to engage in treatment at the 3-month follow-up (60.2% vs 34.4%) in comparison to control. |
| Lin Miller et al (110) | 2017 | Qualitative study | HIV | United States | Community = "high risk youth between the ages of 13 and 24" and focused on specific geographies within each city in which the coalitions were operating. | Community mobilisation through network building and vulnerable group representative in governance arrangements for research and service delivery | Community coalition leadership, collaborative synergy, capacity building, and local community context influence coalitions’ ability to successfully implement HIV-related structural change. Prevention objectives were the most common (n = 205; 36.6%), followed by objectives to build local capacity (n = 160; 28.6%), link HIV-infected youth to medical care (n = 111;19.8%), and improve access to HIV testing (n = 84; 15.0%). |
| Magidson et al (109) | 2022 | Mixed-methods study | HIV | South Africa | Not defined | Peer-to-peer support through mainstream health service delivery | A peer model for reducing substance use disorder stigma among patients and healthcre workers within community-based HIV care teams in found to be promising.75% of stakeholders had high stigma towards a patient with substance use disorder, yet 90% had low stigma when in recovery for at least 2 years. |
| Mayo-Wilson et al (106) | 2020 | Feasibility study | HIV | United States | Not defined | Microenterprise intervention | 82% of economically-vulnerable African-American young people who participated in a trial of 20-week microenterprise intervention found the inclusion of HIV prevention education during sessions was also “very helpful”. |
| Moore et al (111) | 2022 | Quasi-experimental (e.g. time series analysis) | HIV | Multiple | Not defined | Multiple, spanning risk communication and capacity building through network development, promotion of self-care and other measures. | A culturally specific, theory-based group-level program, Brothers Building Brothers by Breaking Barriers (B6), was highly acceptable and participants expressed high levels of satisfaction with the program, although no statistically significant differences between the program and comparison. |
| Muriisa and Jamil (58) | 2011 | Cohort study | HIV | Uganda | Not defined | Social capital generation | Social capital generated through interactions in organizations like TASO and PTC/PLI has increased knowledge sharing and awareness about HIV/AIDS, and reduced stigma and discrimination, allowing people to openly talk about the disease and share experiences. This has helped reduce social isolation. Overall, involvement with one or both of the social mobilisation groups described has improved participants overall quality of life. |
| Mwai et al (33) | 2013 | Systematic review | HIV | Multiple | CHW = any health worker who performs functions related to health care delivery; has trained in some way in the context of the intervention; and has no formal professional or paraprofessional certificate or degree in tertiary education | Community health workers | CHWs enhance the reach, uptake and quality of HIV services, as well as the dignity, quality of life and retention in care of people living with HIV. Clinical outcomes appeared not to be compromised, with no differences in virologic failure and mortality comparing patients in communities or clinics. |
| Newman et al (36) | 2022 | Narrative review | HIV | Multiple | Not defined | Peer-to-peer interventions | The review findings suggest benefits as well as challenges for multicomponent peer education interventions (88% of the studies reviewed) in reaching young key populations and effecting positive changes in knowledge, attitudes, behavioural intentions, and access to services that support HIV prevention and sexual health, with mixed evidence on effecting behaviour change. |
| Remme et al (40) | 2014 | Systematic review | HIV | Multiple | Community engagement "takes into account and addresses the different needs of women/girls and men/boys in its design, or explicitly aims to redress existing inequalities between the sexes." | Multiple, including peer support, community mobilisation activities, micro-enterprise services, poverty reduction and social protection work including cash transfers | Gender empowerment community mobilisation for female sex workers is found to be promising cost-effective intervention for HIV prevention, particularly couple counselling for the prevention of vertical transmission; gender empowerment, community mobilization, and female condom promotion for female sex workers; expanded female condom distribution for the general population; and post-exposure HIV prophylaxis for rape survivors. |
| Sevelius et al (60) | 2022 | Randomised controlled trial | HIV | United States | Not defined | Peer-to-peer intervention | Healthy Divas, an individual-level intervention to increase healthcare empowerment and gender affirmation to improve engagement in HIV care showed short-term efficacy to improve engagement in HIV care among transgender women. At 6 months, intervention participants had over twice the odds of being in a higher HIV care engagement than those in the control arm (aOR = 2.17; 95% CI: 1.06 to 4.45; P = 0.04). |
| Simms et al (62) | 2022 | Randomised controlled trial | HIV | Zimbabwe | Not defined | Peer-to-peer intervention | Problem solving therapy (PST) training for Community Adolescent Treatment Supporters (CATS) did not add to the benefit of peer support in reducing virological nonsuppression but led to improved symptoms of Common Mental Diseases (CMD) and depression compared to standard Zvandiri care among adolescents living with HIV in Zimbabwe (prevalence of nonsuppression 14.7% in the Zvandiri-PST arm versus 11.9% in the Zvandiri arm; AOR = 1.29; 95% CI 0.68, 2.48; p = 0.44). |
| Wilson et al (41) | 2019 | Randomised controlled trial | HIV | United States | Community defined by population. | Peer-to-peer intervention | A strengths-focused HIV prevention program among high-risk heterosexual Black men resulted in reduced sexual risk behavior, and the program was acceptable for administration in partnership with barbershops. The behaviour change was associated with a greater likelihood of no condomless sex (64.4%) than control group participation (54.1%; adjusted odds ratio = 1.61; 95% confidence interval = 1.05, 2.47). |
| UNDP (113) | 2012 | Guidance document | HIV | India | Not defined | Capacity building through community-based organisations | Mobilizing social capital resources for HIV care support increased in scales of medication taking adherence (p = 0.002, FU-1; p = 0.011, FU-2), self-efficacy (p = 0.042; FU-1), and outcome expectancies (p = 0.016, FU-2). Among persons not on ART, HIV Medication Readiness scale scores increased at FU-1 (p = 0.032) but became attenuated at FU-2. |
| WHO (114) | 2018 | Narrative review | Multiple | Multiple | Risk communication = the real-time exchange of information, advice and opinions between experts, community leaders, officials and the people who are at risk and is an integral part of any emergency response | Risk communication | Guidance document focused on building trust and improving community engagement. Results not described. |
| Chiam et al (115) | 2022 | Systematic review | Multiple | Multiple | Community engagement = relationship-building that is required to facilitate the work of pandemic planning with local communities before an outbreak occurs. | Multiple, spanning a full spectrum from risk communication through to community involvement and collaboration initiatives. | Clarity is needed regarding definitions of community. Instrumental and relational aspects to community engagement should be recognised, especially for groups traditionally excluded from decision-making processes. |
| Mohammadpour et al (116) | 2021 | Scoping review | Multiple | Multiple | Not defined | Multiple spanning risk communication, community engagement and other measures | Risk communication and community engagement activities including measures to promote personal and environmental hygiene, behaviour change interventions and communication via social media platforms may be effective in promoting pandemic preparedness and response. |
| Cummings et al (117) | 2019 | Case-control study | Multiple | Sierra Leone | Not defined | Community based communicable disease surveillance | A participatory, community-based health information system using CHWs to collect morbidity and mortality data showed evidence of moderate effect in improving household health behaviours (infant and young child feeding, p=0.002) and some care-seeking behaviours (contraceptive use, p=0.047) but not to a significant degree on wider preparedness measures. |
| Obregon et al (118) | 2020 | Commentary | Multiple | Multiple | Not defined | Risk communication and community outreach | Specific communication and social and behaviour plans are need to set out vaccination priorities, generate vaccine demand, address vaccine hesitancy and mitigate risks associated with adverse events. These are likely to include "social listening" approaches (i.e. monitoring online discussions on immunisation) to better understand people's concerns and expectations and principal points of misinformation to inform countering strategies. |
| Osborne et al (53) | 2021 | Systematic review | Multiple | Multiple | Multiple definitions explored drawing on published literature | Multiple, ranging from risk communication, through consultation to community mobilisation and community engagement in preparedness planning | The success of community engagement is shaped by pre-existing vulnerabilities including local historical and structural factors, and trust in the health system. To be effective, community engagement strategies need to take account of a full spectrum of activities including involvement in decision-making, planning, design, delivery and evaluation of services. |
| Schwartz and Yen (119) | 2017 | Narrative review | Multiple | Taiwan | Not defined | Multiple, but focusing on the role of neighbourhood committees within a "whole of society" approach to preparedness and response | Using a whole-of-society approach to pandemic preparedness and response enhanced cooperation between state, local government and non-state institutions, particularly neighbourhood committees, and resulted in a strengthened, holistic epidemic preparedness and response infrastructure. |
| Collins et al (10) | 2023 | Narrative review | Multiple | Multiple | Not defined | Multiple, spanning risk communication, the use of community leaders as trusted intermediaries, CHWs and others | The intersection between HIV response and pandemic preparedness encompasses various key areas such as surveillance, supply chain, healthcare, community engagement, research, and leadership, offering strategic opportunities to enhance preparedness by integrating existing inclusive and resilient health programs, discouraging the creation of new isolated initiatives. |
| Cook and Seymour (120) | 2013 | Commentary | Multiple | Multiple | Not defined | Multiple but with a primary focus on risk communication and community outreach | Despite the vital role of community ownership and empowerment in the HIV response, uncertainties remain about the relationship between community engagement and HIV outcomes, calling for better understanding and support. The commentary reflects a broader commitment to translate evidence into policies including generating more evidence on health impact pathways and quantifying intervention benefits and costs. |
| Ernawati et al (121) | 2020 | Narrative review | Multiple | Multiple | Not defined | Community health workers | The empowerment model of HIV/AIDS cadres is associated with reducing the stigma and quality of life of women with HIV/AIDS. The cadres offer support to express feelings of pressure, facilitate the safe disclosure of HIV status, build networks of friendship, socialize and provide emotional support so that they become empowered. |
| Iyiani et al (122) | 2011 | Qualitative study | Multiple | Nigeria | Not defined | Multiple including risk communication and community engagement, community mobilisation and capacity building in partnership with community based organisations | Community-based approaches involve recognizing and leveraging the insights and strengths of marginalized local populations, along with diverse stakeholders including international agencies, local NGOs, traditional healers, and cultural groups, to collaboratively address HIV/AIDS vulnerabilities and structural factors in Ajegunle, thereby enhancing prevention efforts at the grassroots level. |
| McGowan et al (123) | 2022 | Systematic review | Multiple | Multiple | Exclusive focus on community-based disease surveillance – defined as “the systematic detection and reporting of events of public health significance within a community by community members” | See left | Success of community-based surveillance (CBS) seen to depend on community acceptance of CBS workers, their motivation to act, quality of supervision and training, approaches to communication and engagement, and wider factors such as the extent of integration with other surveillance systems. |
| Olowu (124) | 2015 | Mixed-methods study | Multiple | Multiple | Not defined | Faith-based approaches to community engagement and empowerment, using a combination of network development, coalition building, trust-building and other measures | Faith-based organizations are contributing energy, expertise, and experience to the global commitment to advance universal access to HIV prevention, treatment, and support, despite challenges, such as the tendency to interpret HIV in religious terms, their general inability to discuss sexuality, and the need to overcome resource and organizational barriers. |
| Wroe et al (125) | 2021 | Randomised controlled trial | Multiple | Malawi | CHWs = "community members outside of the formal nursing or medical profession who are trained to perform tasks ranging from health education, screening for diseases/case finding, referral and accompaniment to care, and sometimes delivery of basic health services." | Community health workers | Expanding an existing HIV and tuberculosis (TB) disease-specific CHW programme into a polyvalent, household-based model results in effective health coverage by reducing default rates from chronic care by 20% , improving uptake of ANC by approximately 30% and paediatric malnutrition case finding declined by 10% |
| Abramowitz et al (126) | 2018 | Systematic review | PHEIC | Multiple | Not defined | Multiple, spanning health communications, community engagement and social mobilisation | Integration of community resources is important for supporting real-time data collection and response integration in epidemic contexts. New techniques are needed for social mobilisation and community engagement in support of this and other preparedness and response objectives. |
| Armstrong-Mensah and Ndiaye (34) | 2018 | Narrative review | PHEIC | Multiple | Community engagement = the process of working collaboratively with and through groups of people affiliated by geographic proximity, special interest, or similar situations to address issues affecting the well-being of those people. | Multiple but a particular focus on the role of community-based surveillance | Community based surveillance complemented facility-based surveillance in community settings in Cambodia, and supporting improved mortality reporting in Chad |
| Barker et al (127) | 2020 | Qualitative study | PHEIC | Liberia | Community engagement = the involvement of communities in decision-making and in the planning, design, governance and delivery of services aimed at improving population health and reducing health inequalities | Multiple, spanning risk communication via different modalities through community engagement to co-development of interventions. | Communities need to be viewed as active participants in health response efforts - some engagement modalities (e.g. community based surveillance) are perceived as more meaningful than others by community members. Trust and community buy-in are both essential for the success of engagement efforts. |
| Bouye et al (128) | 2009 | Commentary | PHEIC | United States | Not defined | Multiple, spanning: community mobilization and partnerships, culturally specific emergency communications planning, culturally specific education and training programs, inclusion of community members as partners, and other modalities. | Effective community engagement and adherence to disease control measures depends on partnership and inclusion in decision-making, culturally-specific education and emergency preparedness approaches, and evidence-based measurement and evaluation approaches, among others. |
| Frimpong and Paintsil (129) | 2023 | Scoping review | PHEIC | Multiple | Community = “group of individuals bound by a geographical boundary, sharing social, traditional or economic interests”  Community engagement = "how organisations and individuals work collaboratively with communities to achieve a collective vision” | Multiple, spanning information sharing and other communication approaches, community-based surveillance, community-based treatment modalities and other measures. | Measures such as education and community-based surveillance can improve case ascertainment for Ebola, improve knowledge and behaviours in terms of prevention and control. Engagement of community members and particularly local leaders in building trust, was important for ensuring positive outcomes from interventions. |
| Kiser and Lovelace (130) | 2019 | Mixed-methods study | PHEIC | United States | Not defined | Faith-based approaches to community engagement and empowerment, using a combination of network development, coalition building, trust-building and other measures to promote uptake of seasonal flu vaccination | The use of intermediary organisations boosted information sharing and distribution of best practice through multiple channels to support pandemic preparedness. Trust and commitment to a shared vision were important for the success of the partnership model outlined in this piece. |
| Lwin et al (28) | 2018 | Qualitative study | PHEIC | Singapore | Not defined | Risk communication via social media | Facebook provided a versatile risk communication tool and was used in different ways across pandemic phases in Singapore. Risk messages (focused on core information about the pathogen and symptoms) generated the greatest engagement across all phases although measures of engagement used in this study were crude. |
| Mase et al (131) | 2017 | Mixed-methods study | PHEIC | United States | Not defined | Risk communication | Risk communication was supported through multiple channels including social media, alternative language productions (for Spanish-speakers) and through cooperative agreements with schools to support dissemination. |
| Masotti et al (132) | 2013 | Narrative review | PHEIC | Canada | Not defined | Community preparedness planning | Community Pandemic Influenza Plans, incorporating all local stakeholders, can be important in delineating agreed-upon roles and responsibilities. |
| Mayhew et al (4) | 2021 | Narrative review | PHEIC | DR Congo | Not defined | Multiple, spanning risk communication, community engagement and trust-building initiatives, the use of trusted intermediaries and others | Communities were engaged in Ebola response in the 2018-20 outbreak in DRC to understand their perceptions but did not have a role in decision-making or leadership. Attention to trust and power dynamics are key in understanding the role(s) that communities can play in pandemic preparedness but are rarely documented. Many different approaches can promote better engagement including the use of community liaison teams, partnerships, engaging community leaders, community-based surveillance and others |
| Meyer et al (133) | 2018 | Narrative review | PHEIC | United States | Not defined | Risk communication and community outreach | Health facilities that opened their Ebola treatment centres for public viewing prior to the epidemic had better relationships with neighbouring communities were better able to treat those affected. Intensive communication is needed in the pre-epidemic phase to improve awareness and build trust at community level. |
| Miller et al (134) | 2018 | Mixed-methods study | PHEIC | Multiple | Not defined, but describes community health workforce as including community health workers (CHWs, traditional birth attendant (TBA), and community health committees (CHC). | Community health workers | CHWs were able to continue delivering services during the West African Ebola outbreak and to deliver risk-reduction interventions better than outsiders. Future preparedness can be supported by better delineation of CHWs roles and responsibilities and consistent support (financial, material, other) throughout. |
| Ndiaye et al (135) | 2014 | Mixed-methods study | PHEIC | Chad | Not defined | Social mobilisation through engagement of trusted community leaders | Polio vaccination uptake among nomadic populations in this study increased sharply pre-to-post campaign, as a result of a multi-pronged strategy in which participation of nomadic community leaders featured heavily, as well as the use of social mobilisers for vaccination and targeted activities during market days when many community members met. Small financial incentives were used to promote community leader involvement. |
| Nsubuga et al (136) | 2021 | Mixed-methods study | PHEIC | Uganda | Not defined | Multiple spanning risk communication, community outreach and engagement via trusted leaders, to community-based disease surveillance drawing on volunteer health workers. | Involvement of religious, cultural and opinion leaders locally all contributed to improved uptake of risk messaging for viral haemorrhagic fevers (based on qualitative assessment by participants in the study) and should form a central plank of future preparedness and response efforts. |
| Olu et al (137) | 2016 | Narrative review | PHEIC | Sierra Leone | Not defined | Community involvement through the participation of trusted leaders in decision-making around preparedness and response | Paramount chiefs from local areas were engaged in response coordination at district level but community opposition to response activities remained strong because of a perception that treatment centres where in reality places in which most admitted patients died. Future pandemic preparedness activities should incorporate anthropological work to understand local cultural norms and ways in which these may influence engagement activities. |
| Simen-Kapeu et al (138) | 2021 | Mixed-methods study | PHEIC | Liberia | Not defined | Community health workers to support delivery of integrated community case management | Establishing a coordination mechanism and leveraging partnership support; using a systems approach to better inform policy shifts; strengthening community engagement; and conducting evidence-based planning to inform policy-makers are key learnings in the community health policy development for strengthening health system resilience. |
| Skrip et al (54) | 2020 | Mixed-methods study | PHEIC | Sierra Leone | Not defined | Multi-dimensional programme featuring community health volunteers to support service delivery, participation of community members in programme governance, and the development of community preparedness plans | Community-based development of locally feasible, locally owned action plans, with the support of community mobilisers, has potential to foster behaviour change in outbreak settings. Significant associations were observed between unsatisfied needs categories and both safe burials (relative risk [RR] 0·86, 95% credible interval [CrI] 0·82–0·91) and fewer prompt referrals to treatment (RR 0·76, 0·70–0·83). Compared with triggering visits by community mobilisers, follow-up visits were associated with higher numbers of prompt referrals (RR 1·40, 95% CrI 1·30–1·50) and safe burials (RR 1·08, 1·02–1·14). |
| Wilkinson et al (48) | 2017 | Narrative review | PHEIC | Multiple | Problematises community engagement as "too vague, too variable in its application and definitions to be of much utility as an analytical tool". Recognises broad definitions of community focusing on geography, specific population groups or other boundaries. | Trust-building through involvement of recognised community leaders | Within the West Africa Ebola Response, building trust and facilitating community independent learning, externally instigated social mobilization and community engagement efforts contributed to understanding social dynamics is essential to designing robust interventions and should be a priority in public health and emergency planning. |
| WHO | 2018 | Guidance document | PHEIC | Multiple | Not defined. | Risk communication and community engagement | Guidance document focusing on risk communication and community response for the Ebola response in the Democratic Republic of the Congo. No reported results. |
| WHO (139) | 2022 | Guidance document | PHEIC | Multiple | Community engagement = "collaborative process that involves people in understanding the risks they face and includes communities in developing health and response practices that are acceptable and workable for them. The goal of community engagement is to empower communities and to develop shared leadership throughout the emergency response cycle." A community is a group of people connected by common characteristics, such as geographic location, age, gender, profession, ethnicity, faith, shared vulnerability or risk, or shared interests and values. Communities can be virtual, and people can belong to many at once, each with different priorities and perceptions. Vulnerable communities are those who are marginalized or who have limited access to health and social services and may be worse affected. | Risk communication and community engagement | Interim guidance document on risk communication and community engagement for monkeypox outbreaks. No reported results. |
| IFRC, UNICEF, WHO (140) | 2018 | Guidance document | PHEIC | DR Congo | Risk communication = "real time exchange of information, opinion and advice between frontline responders and people who are faced with the threat of Ebola to their survival, health, economic or social wellbeing"  Community engagement = "mutual partnership between Ebola response teams and individuals or communities in affected areas, whereby community stakeholders have ownership in controlling the spread of the outbreak" | Risk communication and community engagement | Guidance document offering a risk communication and community engagement preparedness and readiness framework for the Ebola response in the Democratic Republic of Congo in North Kivu. No reported results. |
| Singaravelu et al (141) | 2019 | Narrative review | PHEIC | DR Congo | Not defined | Risk communication and community engagement | The deployment of a multidisciplinary team of anthropologists, risk communicators and social mobilizers were integral to ensuring sustained engagement, resulting in more effective Ebola response in Équateur Province, DRC,in 2018 . Streamlined local and global coordination through regular meetings, online platforms and mobile messaging ensured rapid sharing of information between operational response partners. |
| UNICEF, WHO (142) | 2014 | Guidance document | PHEIC | Multiple | Not defined | Multiple, spanning risk communication, community engagement and community mobilisation through messaging via trusted channels | Guidance document on the key messages for social mobilisation and community engagement in intense transmission areas of Ebola virus. No reported results. |

#### S1 References

152. Alberti PM, Lantz PM, Wilkins CH. Equitable Pandemic Preparedness and Rapid Response: Lessons from COVID-19 for Pandemic Health Equity. J Health Polit Policy Law. 2020;45(6):921–35.

153. Coleman JD, Lindley LL, Annang L, Saunders RP, Gaddist B. Development of a framework for HIV/AIDS prevention programs in African American churches. AIDS Patient Care STDS. 2012;26(2):116–24.

154. DeBruin D, Liaschenko J, Marshall MF. Social justice in pandemic preparedness. Am J Public Health. 2012;102(4):586–91.

155. Gilmore B, Ndejjo R, Tchetchia A, De Claro V, Mago E, Diallo AA, et al. Community engagement for COVID-19 prevention and control: A rapid evidence synthesis. BMJ Glob Health. 2020;5(10).

156. International Labour Organization. Addressing stigma and discrimination in the COVID-19 response: Key lessons from the response to HIV and AIDS. 2020;(May):1–2.

157. Jewett RL, Mah SM, Howell N, Larsen MM. Social Cohesion and Community Resilience During COVID-19 and Pandemics: A Rapid Scoping Review to Inform the United Nations Research Roadmap for COVID-19 Recovery. International Journal of Health Services. 2021;51(3):325–36.

158. Kamaka ML, Freitas SM, Marshall SM, Walsh ME, Kamakawiwo’ole S, Miller JM, et al. He ’A’ali’i Kū Makani Mai Au: Developing a Cultural Framework for Advancing COVID-19 Related, Community-informed Health Policies. Hawaii J Health Soc Welf. 2021;80(10):50–6.

159. Khan S, Mishra J, Ahmed N, Onyige CD, Lin KE, Siew R, et al. Risk communication and community engagement during COVID-19. International Journal of Disaster Risk Reduction. 2022;74(May 2020):102903.

160. Liu Z, Lin S, Shen Y, Lu T. Collaborative neighborhood governance and its effectiveness in community mitigation to COVID-19 pandemic: From the perspective of community workers in six Chinese cities. Cities. 2021;116(May):103274.

161. Lohiniva AL, Pensola A, Hyökki S, Sivelä J, Tammi T. COVID-19 risk perception framework of the public: an infodemic tool for future pandemics and epidemics. BMC Public Health. 2022;22(1):1–9.

162. Maunula L. The pandemic subject: Canadian pandemic plans and communicating with the public about an influenza pandemic. Healthcare Policy. 2013;9(SPEC. ISSUE):14–25.

163. McCollum R, Zaizay Z, Dean L, Watson V, Frith L, Alhassan Y, et al. Qualitative study exploring lessons from Liberia and the UK for building a people-centred resilient health systems response to COVID-19. BMJ Open. 2022;12(8):1–13.

164. Murthy BP, Leblanc TT, Vagi SJ, Avchen RN. Going viral: The 3 rs of social media messaging during public health emergencies. Health Secur. 2021;19(1):75–81.

165. O’Grady N, Shaw D, Parzniewski S. People in a pandemic: Rethinking the role of ‘Community’ in community resilience practices. Geoforum. 2022;132(April):32–41.

166. Oliveira M, Braga MF, Bueno A, De Sousa DP, Pigozi PL, Moryia R, et al. Actions during the COVID-19 pandemic to protect the most vulnerable population: what is the potency amid chaos? Health Promot Int. 2022;37(2).

167. O’Sullivan TL, Phillips KP. From SARS to pandemic influenza: the framing of high-risk populations. Natural Hazards. 2019;98(1):103–17.

168. Crouse Quinn S. Crisis and emergency risk communication in a pandemic: a model for building capacity and resilience of minority communities. Health Promot Pract. 2008;9(4 Suppl):18–25.

169. Rela IZ, Ramli Z, Firihu MZ, Widayati W, Awang AH, Nasaruddin N. COVID-19 Risk Management and Stakeholder Action Strategies: Conceptual Frameworks for Community Resilience in the Context of Indonesia. Int J Environ Res Public Health. 2022;19(15).

170. WHO Regional Office for Europe. Strengthening social science capacities in the European Region.
